# Covalent Functionalization of Bioengineered Polyhydroxyalkanoate Spheres Directed by Specific Protein-Protein Interactions

**DOI:** 10.3389/fbioe.2020.00044

**Published:** 2020-02-06

**Authors:** Jin Xiang Wong, Majela Gonzalez-Miro, Andrew J. Sutherland-Smith, Bernd H. A. Rehm

**Affiliations:** ^1^School of Fundamental Sciences, Massey University, Palmerston North, New Zealand; ^2^MacDiarmid Institute for Advanced Materials and Nanotechnology, Victoria University of Wellington, Wellington, New Zealand; ^3^Adaptive Immunity, Biomedical Centre, Lund University, Lund, Sweden; ^4^Centre for Cell Factories and Biopolymers, Griffith Institute for Drug Discovery, Griffith University, Nathan, QLD, Australia

**Keywords:** polyhydroxyalkanoates, polyesters, Tag/Catcher systems, modular functionalization, synthetic biology, biotechnology, polymer science

## Abstract

Bioengineered polyhydroxyalkanoate (PHA) spheres assembled in engineered bacteria are showing promising potential in protein immobilization for high-value applications. Here, we have designed innovative streamlined approaches to add functional proteins from complex mixtures (e.g., without prior purification) to bioengineered PHA spheres directly harnessing the specificity of the SpyTag/SpyCatcher mediated protein ligation. *Escherichia coli* was engineered to assemble PHA spheres displaying the SpyCatcher domain while simultaneously producing a SpyTagged target protein, which was *in vivo* specifically ligated to the PHA spheres. To further demonstrate the specificity of this ligation reaction, we incubated isolated SpyCatcher-coated PHA spheres with cell lysates containing SpyTagged target protein, which also resulted in specific ligation mediating surface functionalization. An even cruder approach was used by lysing a mixture of cells, either producing PHA spheres or target protein, which resulted in specific surface functionalization suggesting that ligation between the SpyCatcher-coated PHA spheres and the SpyTagged target proteins is highly specific. To expand the design space of this general modular approach toward programmable multifunctionalization, e.g., one-pot construction of immobilized multienzyme cascade systems on PHA spheres, we designed various recombinant bimodular PHA spheres utilizing alternative Tag/Catcher pairs (e.g., SnoopTag/SnoopCatcher and SdyTag/SdyCatcher systems). One of our bimodular PHA spheres resulted in simultaneous multifunctionalization of plain PHA spheres in one-step with two differently tagged proteins under *in vitro* and *ex vivo* reaction conditions while remaining functional. Our bimodular PHA spheres also showed high orthogonality with the non-target peptide tag and exhibited decent robustness against repeated freeze-thaw treatment. We demonstrated the utility of these approaches by using a fluorescent protein, a monomeric amylase, and a dimeric organophosphate hydrolase as target proteins. We established a versatile toolbox for dynamic functionalization of PHA spheres for biomedical and industrial applications.

## Introduction

Metabolic pathways often require biochemical processes that are dependent on multiprotein complexes assembled on a variety of biological scaffolds and found in compartments in numerous prokaryotic and eukaryotic organisms (Diekmann and Pereira-Leal, 2013; Cornejo et al., 2014). Artificial organization of immobilized multiprotein complexes, where multiple individual proteins working in consortia to carry out specific tasks have been increasingly considered for development of next-generation biocatalysts (Ren et al., 2019). The exciting approach of creating such biomimetic scaffold structures to localize the active sites of proteins into proximity increase the local concentrations of these active units, further improving function, and robustness of the relevant proteins (Bae et al., 2015; Hwang and Lee, 2019). Precise control of immobilized multiprotein complexes on defined scaffolding architecture also enables efficient substrate directionality (e.g., physical channeling) and shielding of any unstable intermediates from the bulk phase (Siu et al., 2015; Qu et al., 2019). Therefore, there is a growing interest in biomaterials research that aims at developing customizable generic biological scaffolds. With the advances in the field of synthetic biology, it is feasible to employ a bottom-up approach in constructing artificial multifunctional scaffolds, focusing on three components: task-specific functional domains of interest, bioorthogonal immobilization sites, and a generic scaffolding platform.

Bioengineered polyhydroxyalkanoates (PHAs) have been proven as promising scaffolds for one-step *in vivo* protein immobilization. PHAs are polyesters produced in nature by microorganisms and stored in their cytosol under excess carbon and nutrient-deprived conditions. Several bacterial strains can be engineered to allow production and *in vivo* directed self-organization of shell-core like spheres, where surface functionalization of such PHA spheres can be achieved by genetic manipulation of PHA-associated proteins and/or chemical modification after isolation (Parlane et al., 2016b). Notably, this can be achieved by genetic fusion of protein domains of interest to surface-exposed PHA-associated proteins such as the PHA synthase (PhaC). PhaC is an essential enzyme in the microbial synthesis of PHA spheres as it catalyzes polymerization of (*R*)-3-hydroxybutyryl-coenzyme A (CoA) to PHA and remains covalently attached to the PHA polymer chain via the active site cysteine residue as a dimeric protein (Tian et al., 2005). We harnessed the surface-exposed arrangement of *Cupriavidus necator* (formerly *Ralstonia eutropha*) PhaC on bacterial PHA spheres by genetically combining PhaC with a variety of protein domains for uses in therapeutic protein production and purification (Du and Rehm, 2017, 2018), vaccination (Rubio-Reyes et al., 2016; Gonzalez-Miro et al., 2018b), diagnostic tools (Parlane et al., 2016a), and biocatalysis (Rasiah and Rehm, 2009; Blatchford et al., 2012). However, the utilization of PhaC as the docking domain for the surface display of different functional proteins lacks control over some properties, such as surface coverage and orientation of the attached proteins, potential failure in protein folding (e.g., eukaryotic proteins) (Du and Rehm, 2017, 2018), inability to enable post-translational modifications and a lack of control over the amount of immobilized functional proteins. In addition, the direct genetic fusion of functional proteins to PhaC impacted sphere assembly, which influenced production yields and sphere sizes (Rubio-Reyes et al., 2016; Gonzalez-Miro et al., 2018b). Although the PHA sphere display technology has led to multiple successful prototypes, its complex biological assembly enables less control over surface functionalization.

**FIGURE 1:**
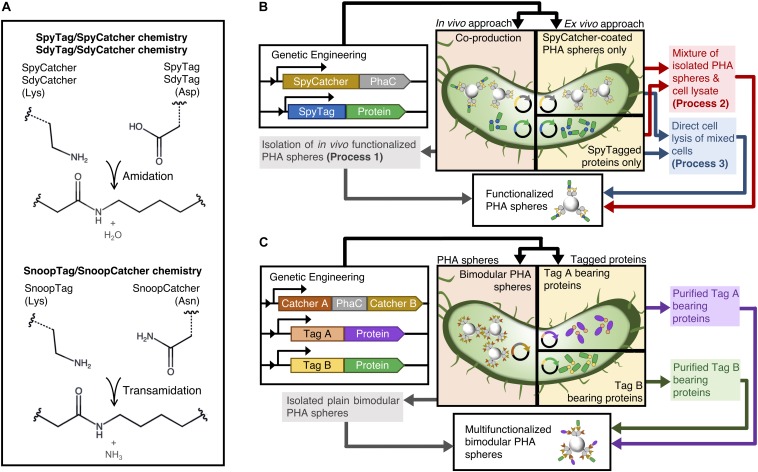
Schematic of modular functionalization of PHA spheres. **(A)** Various Tag/Catcher systems. **(B)** Various one-pot modular functionalization processes established in this study. **(C)** Simultaneous dual functionalization of PHA spheres using combinations of Catcher domains displayed on PHA spheres.

To overcome these limitations, we propose to merge the PHA sphere display technology with the recently developed SpyTag/SpyCatcher chemistry derived from *Streptococcus pyogenes* (Figure 1A; Zakeri et al., 2012). A spontaneous covalent isopeptide bond forms between a lysine residue of the SpyCatcher domain (13 kDa) and an aspartic acid of its pairing peptide SpyTag (13 amino acid residues) in a site-specific manner, without the need of additional reagents nor enzymes at broad ligation conditions (Reddington and Howarth, 2015). The advantageous properties of the SpyTag/SpyCatcher chemistry makes it an excellent protein ligation tool for surface functionalization of various organic and inorganic materials, such as virus-like particles (Brune et al., 2016, 2017; Bruun et al., 2018), protein-based scaffolds (Bae et al., 2018; Choi et al., 2018; Swartz and Chen, 2018; Zhang et al., 2018a), gold nanoparticles (Ma et al., 2018), silica (Zhang et al., 2018b, c), quantum dots (Ke et al., 2018; Brizendine et al., 2019), and crystalline graphene (Tyagi et al., 2018). We recently developed a modular PHA platform using SpyTag/SpyCatcher chemistry, where we successfully showed that purified SpyTagged proteins could ligate to SpyCatcher-coated PHA spheres *in vitro* with decent tunability (Wong and Rehm, 2018). Relatively consistent physicochemical properties of PHA spheres were achieved, regardless of the functional moieties decorating the particulate PHA scaffold, while retaining or enhancing functionality of the immobilized target proteins. This approach allows robust and covalent functionalization of PHA spheres without being constrained by the direct genetic fusion method.

In this study, we first aim to streamline this modular functionalization approach using different process steps, testing whether SpyTagged proteins could be ligated to SpyCatcher-coated PHA spheres without the need of purifying soluble tagged proteins by using one *in vivo* and two *ex vivo* functionalization processes namely processes 1-3 (Figure 1B and Supplementary Figures S1–S3). Thereby, we are not only avoiding purification of individual components but also using a single lysis step, which further improves time and cost savings. Functionalization occurs during the cell lysis step, and we propose that the immediate release of target components from the bacterial cells leads to specific covalent ligation between PHA sphere and target protein *ex vivo* during the cell disruption process. Nevertheless, although our previous study presented that SpyCatcher-coated PHA spheres able to co-localize different SpyTagged proteins, the sequential and reactant ratio-dependent strategies proposed could impose manufacturing burdens (Wong and Rehm, 2018). Therefore, to expand the concept of modularity beyond our initial studies based on SpyTag/SpyCatcher chemistry solely, we attempted to incorporate two non-cross reacting directed peptide-protein pairs with PHA sphere technology to construct an efficient bimodular polymeric scaffolding platform (Figure 1C). In addition to the PHA sphere technology-compatible SpyTag/SpyCatcher chemistry pair, we further considered alternative orthogonal Tag/Catcher pairs, namely SdyTag/SdyCatcher derived from *Streptococcus dysgalactiae* (Tan et al., 2016) and SnoopTag/SnoopCatcher derived from *Streptococcus pneumoniae* (Veggiani et al., 2016), for the construction of our bimodular polymeric scaffolding platform (Figure 1A). We also genetically fused different covalent peptide tags to the N-terminus of *Aequorea victoria* green fluorescent protein (GFP) and *Bacillus licheniformis* α-amylase (BLA) to allow site-specific protein ligation to the Catcher domains displayed on PHA spheres.

## Materials and Methods

### Bacterial Strains, Genetic Manipulation, and Culture Conditions

All the bacterial strains, plasmids, and primers used in the current study are listed in Supplementary Tables S1-S3, respectively. The primers used for genetic manipulation were ordered from Integrated DNA Technologies (San Diego, CA, United States). DNA extraction and genetic engineering procedures were performed as described (Sambrook et al., 1989). For plasmid harboring and cloning, *Escherichia coli* XL1-Blue (Stratagene, La Jolla, CA, United States) was grown overnight (16 h) in Luria-Bertani, Lennox medium (LB-Lennox) at pH 7.5 under 37°C and 200 rpm. If required, ampicillin (100 μg/mL), chloramphenicol (50 μg/mL) and kanamycin (50 μg/mL) were introduced. Detailed plasmid construction strategies are described in the Supplementary Material. Positive constructed clones were transformed into the appropriate competent *E. coli* BL21(DE3) cells (Invitrogen, Carlsbad, CA, United States), and competent *E. coli* BL21(DE3) cells harboring plasmid pMCS69 for production of soluble proteins and PHA spheres, respectively. Plasmid pMCS69 allows the synthesis of the precursor *R*-3-hydroxybutryl-coenzyme A (CoA), which is essential to biosynthesis of PHA spheres.

### Polyhydroxyalkanoate (PHA) Sphere and Soluble Protein Production

An overnight culture of the production strains was inoculated at a 100-fold dilution into fresh LB-Lennox medium containing appropriate antibiotics supplemented with 1% (w/v) glucose. The medium was cultured at 37°C and 200 rpm until an OD600 of ∼0.6 was achieved. After that, 1 mM isopropyl β-D-1-thiogalactopyranoside (IPTG) was added to the cultures to induce protein production. Growth media were harvested after 24 h incubation at 30°C and 48 h at 25°C, respectively, for soluble protein and PHA sphere production.

### Protein Analysis

All fusion proteins were analyzed by sodium dodecyl sulfate-polyacrylamide gel electrophoresis (SDS-PAGE) as described elsewhere (Laemmli, 1970). Briefly, protein samples were separated in 10% (v/v) polyacrylamide separating gels with 4% (v/v) polyacrylamide stacking gels. The molecular mass of the samples was estimated using GangNam-STAIN prestained protein ladder (iNtRON Biotechnology, Seongnam, South Korea). SDS-PAGE gels were stained with 0.05% (w/v) Coomassie brilliant blue R-250 dye, 50% (v/v) ethanol and 10% (v/v) acetic acid for 30 min and then destained in 50% (v/v) ethanol and 10% (v/v) acetic acid for 2 h. Images of polyacrylamide gels were taken using Gel Doc XR + system (Bio-Rad Laboratories, Hercules, CA, United States).

### Protein Quantification

Protein concentration was determined by measuring the band intensity from SDS-PAGE gels by densitometric analysis using Image Lab 5.2.1 software (Bio-Rad Laboratories, Hercules, CA, United States) and comparing the value to a standard curve prepared from known concentrations of bovine serum albumin (BSA) standard as described elsewhere (Hay et al., 2014).

### Peptide Mass Fingerprinting

Purified protein bands from the SDS-PAGE gel were excised and subjected to tryptic hydrolysis as described (Shevchenko et al., 2006). The resulting extracted tryptic peptide samples were then analyzed by liquid chromatography-tandem mass spectrometry (LC-MS/MS).

### Isolation of Plain Catcher Domain-Coated PHA Spheres and *in vivo* Functionalized Catcher Domain-Coated PHA Spheres (Process 1)

The cell pellets harvested by centrifugation (8,000 *g* at 4°C for 20 min) were resuspended and washed with 10 mM Tris–HCl (pH 7.5) prior to cell lysis. Washed cells were mechanically disrupted by passing through a M-110P microfluidizer (Microfluidics, Westwood, CA, United States) at least three passes (1500 bar). After cell lysis, PHA spheres were recovered by centrifugation (9,500 *g* at 4^*o*^C for 30 min). Recovered PHA spheres were then washed at least three times and resuspended in PHA storage buffer (50 mM Tris–HCl, 20% v/v ethanol, pH 7.5) and stored at 4°C for further analysis.

### Isolation and *ex vivo* Functionalization of Catcher Domain-Coated PHA Spheres (Process 2)

Plain Catcher domain-coated PHA spheres were isolated as described in Process 1 above. Meanwhile, the cell pellets containing tagged soluble proteins were washed in 10 mM Tris–HCl (pH 7.5) once before cell lysis. Washed cell pellets were resuspended to 10% cell slurry in 50 mM Tris–HCl (pH 7.5) and mechanically disrupted. After cell lysis, the whole-cell lysate was centrifuged (9,500 *g* at 4°C for 1 h) to discard the insoluble cellular debris. The supernatant was filtered through a 0.22 μm cellulose acetate membrane filter (ReliaPrep, Ahlstrom-Munksjö, Helsinki, Finland). The resulting *E. coli* cleared lysate containing tagged soluble proteins was then mixed with the plain Catcher domain-coated PHA spheres for 24 h at 25°C. After that, the functionalized PHA spheres were recovered by centrifugation (9,500 *g* at 4°C for 30 min) and washed at least three times with 50 mM Tris–HCl (pH 7.5). The functionalized PHA spheres were then resuspended in PHA storage buffer (50 mM Tris–HCl, 20% v/v ethanol, pH 7.5) and stored at 4°C for further analysis.

### Isolation and *ex vivo* Functionalization of Catcher Domain-Coated PHA Spheres (Process 3)

Growth media were harvested after 24 h incubation at 30°C. The cultures were centrifuged (8,000 *g* at 4°C for 20 min) and washed in 10 mM Tris–HCl (pH 7.5) once before cell lysis. Washed cell pellets containing tagged soluble proteins and Catcher domain-coated PHA spheres were mixed at a mass ratio of 1:1 and resuspended to 10% cell slurry which was sonicated through 10 s pulses for 5 min at an output setting of 2.5 using a Virsonic 600 sonicator (SP Scientific, Gardiner, NY, United States). After cell lysis, functionalized PHA spheres were recovered by centrifugation (9,500 *g* at 4°C for 30 min) and washed at least three times with 50 mM Tris–HCl (pH 7.5). The washed PHA sphere pellets were then resuspended in PHA storage buffer (50 mM Tris–HCl, 20% v/v ethanol, pH 7.5) and stored at 4°C for further analysis.

### Isolation and Purification of Tagged Soluble Protein

The cell pellets recovered by centrifugation (8,000 *g* at 4°C for 20 min) were washed with 20 mM Tris–HCl (pH 7.5) at least once prior to cell lysis. Washed cell pellets were resuspended in 1 × protein lysis buffer (50 mM Tris–HCl, 300 mM NaCl, 40 mM imidazole, pH 7.5) to 10% cell slurry and mechanically disrupted. The whole-cell lysate was subjected to centrifugation (9,500 *g* at 4°C for 1 h) after cell lysis to remove the cellular debris. The resulting cleared cell lysate was filtered through a 0.22 μm polyethersulfonate membrane filter in a bottle-top vacuum filter system (Corning Inc., Corning, NY, United States). Subsequently, the filtered supernatant was pumped through a 5 mL nickel-nitrilotriacetic acid (Ni-NTA) chromatography column (HisTrap HP, GE Healthcare, Buckinghamshire, United Kingdom) at 5 mL/min using a peristaltic pump (LongerPump, Longer Precision Pump, Hebei, China). At least 5 column volumes of protein wash buffer (50 mM Tris–HCl, 300 mM NaCl, 50 mM imidazole, pH 7.5) were pumped into the Ni-NTA column at 5 mL/min to remove the unbound and non-specifically bound proteins. The immobilized proteins were eluted from the resins by the addition of at least 5 column volumes of protein elution buffer (50 mM Tris–HCl, 300 mM NaCl, 500 mM imidazole, pH 7.5) into the Ni-NTA column. The eluates were concentrated and desalinated using a centrifugal concentrator (Vivaspin 20, GE Healthcare, Buckinghamshire, United Kingdom). The concentrated eluates were then stored at 4°C for further analysis.

### *In vitro* Functionalization of Various Catcher Domain-Coated PHA Spheres

To functionalize various Catcher-domain coated PHA spheres they were mixed and incubated with tagged *A. victoria* green fluorescent protein (GFP), *Agrobacterium radiobacter* organophosphohydrolase (OpdA), or *Bacillus licheniformis* α-amylase (BLA) at a Catcher-Tag reactant ratio of 1:5 (50 mM Tris–HCl, pH 7.5) at 4°C under constant rotary shaking overnight at 20 rpm. The PHA spheres were washed at least three times (50 mM Tris–HCl, pH 7.5) to remove the unbound soluble proteins and stored at 4°C for further use and analysis.

### Compositional Analysis of PHA Spheres

Approximately 75 mg of lyophilized PHA spheres were subjected to methanolysis as described elsewhere (Braunegg et al., 1978). The organic layer of all samples was recovered, filtered, and further analyzed by gas chromatography-mass spectroscopy (GC-MS) using poly-(*R*)-3-hydroxybutyrate (PHB) as a standard.

### Scanning Electron Microscopy (SEM) and Transmission Electron Microscopy (TEM) Analysis

PHA spheres were processed for SEM and TEM by the Manawatu Microscopy and Imaging Centre (MMIC, Massey University, Palmerston North, New Zealand). SEM micrographs of the processed samples were imaged using an FEI Quanta 200 Environmental Scanning Electron Microscope, and TEM micrographs of the processed samples were imaged using an FEI Tecnai G2 BioTwin Transmission Electron Microscope.

### Particle Size Distribution (PSD) Measurement

The particle size distribution of the PHA spheres was determined by dynamic light scattering (DLS) analysis using the Mastersizer 3000 laser diffraction sphere size analyzer (Malvern Instruments, Malvern, United Kingdom) at room temperature (25°C) with a helium-neon (He-Ne, λ = 632.8 nm) laser. The PHA sphere samples were prepared in 0.1% (w/v) of wet PHA spheres in storage buffer (50 mM Tris–HCl, 20% (v/v) ethanol, pH 7.5).

### Fluorescence Microscopy Analysis

Soluble or immobilized GFP in 50 mM Tris–HCl, pH 7.5 were evaluated for their fluorescence signals. Fluorescence microscopy images of the samples were taken using an Olympus BX51 Fluorescent Light Microscope (Olympus Optical, Tokyo, Japan) at 100× magnification using MicroPublisher 5.0 color CCD camera and QCapture Pro 6.0 application software (QImaging, Surrey, BC, Canada).

### Qualitative Starch Degradation Screen

Enzymatic activity of soluble, or immobilized, BLA was qualitatively verified using starch agar plates (Rasiah and Rehm, 2009). Briefly, 1% starch agar was prepared by dissolving 1% (w/v) starch and 1.5% (w/v) agar with 50 mM Tris–HCl, 300 mM NaCl buffer (pH 7.5) prior to autoclaving. All samples were incubated at 37°C up to 24 h on the surface of the starch agar plates. After that, the starch agar plates were washed with deionized water once before subjected to Lugol’s iodine staining for 5 min at room temperature (25°C). Lugol’s iodine was drained and washed off with deionized water again prior to imaging.

### Organophosphohydrolase Functionality Assay

Enzymatic activity of soluble, or immobilized OpdA (50 mM Tris–HCl, pH 7.5) with negative controls was measured using an assay mixture of 250 μM coumaphos dissolved in a modified reaction buffer (50 mM Tris–HCl, 20% (v/v) methanol, pH 7.5) at fixed concentration of soluble, or immobilized OpdA (0.5 μM) (Harcourt et al., 2002). Quantification of liberated chlorferon from coumaphos was determined by FluoroMax^®^-4 and a Jobin Yvon MicroMax 384 microwell-plate reader controlled by FluoEssence version 3.5 (HORIBA Scientific, Kyoto, Japan) at excitation and emission wavelengths of 355 and 450 nm, respectively. Samples were loaded in an assay mixture and assayed at room temperature (25°C) for up to 2 h at 10 min intervals.

### Heat-Cooling Cycle Stability

A suspension of plain SnoopCatcher-PhaC-SpyCatcher fusion protein-coated PHA spheres (NPP-S) in 50 mM Tris–HCl (pH 7.5) was subjected to up to five cycles of incubation at 95^*o*^C for 15 min and cooled down with an ice bath at 4^*o*^C for 15 min. All the samples were washed with 50 mM Tris–HCl (pH 7.5) three times before mixed with tagged proteins at a Catcher-Tag reactant ratio of 1:5 (50 mM Tris–HCl, pH 7.5) at 4°C under constant rotary shaking overnight at 20 rpm. The functionalized Catcher domain-coated PHA spheres were washed at least three times (50 mM Tris–HCl, pH 7.5) to remove the unbound soluble tagged proteins and stored at 4°C before subjected to SDS-PAGE analysis and blue light exposure.

### Freeze-Thaw Cycle Stability

A suspension of plain NPP-S in 50 mM Tris–HCl (pH 7.5) was subjected to up to five freeze-thaw cycles, where the samples were frozen at -20^*o*^C for overnight and thawed at 4^*o*^C for 8 h (50 mM Tris–HCl, pH 7.5). Then, the thawed samples were washed at least three times (50 mM Tris–HCl, pH 7.5). The washed Catcher domain-coated PHA spheres were incubated with tagged proteins at 4°C overnight under constant rotary incubation at 20 rpm using a Catcher-Tag reactant ratio of 1:5. All the functionalized Catcher domain-coated PHA spheres were washed at least three times (50 mM Tris–HCl, pH 7.5) to remove the unbound soluble tagged proteins and stored at 4°C before subjected to SDS-PAGE analysis and blue light exposure.

## Results

This study presents several innovative approaches to further expand the design space of PHA spheres as an advanced platform technology to manufacture functional materials of biomedical and industrial uses. We will first report the results that support the efficiency of the proposed streamlined processes to functionalize our modular SpyCatcher-coated PHA spheres. Then, we detail the utility of multiple orthogonal protein ligation systems toward the construction of bimodular PHA spheres, which ultimately enable simultaneous dual functionalization of our bimodular PHA spheres.

### Production and Characterization of SpyCatcher-Coated PHA Spheres

**FIGURE 2:**
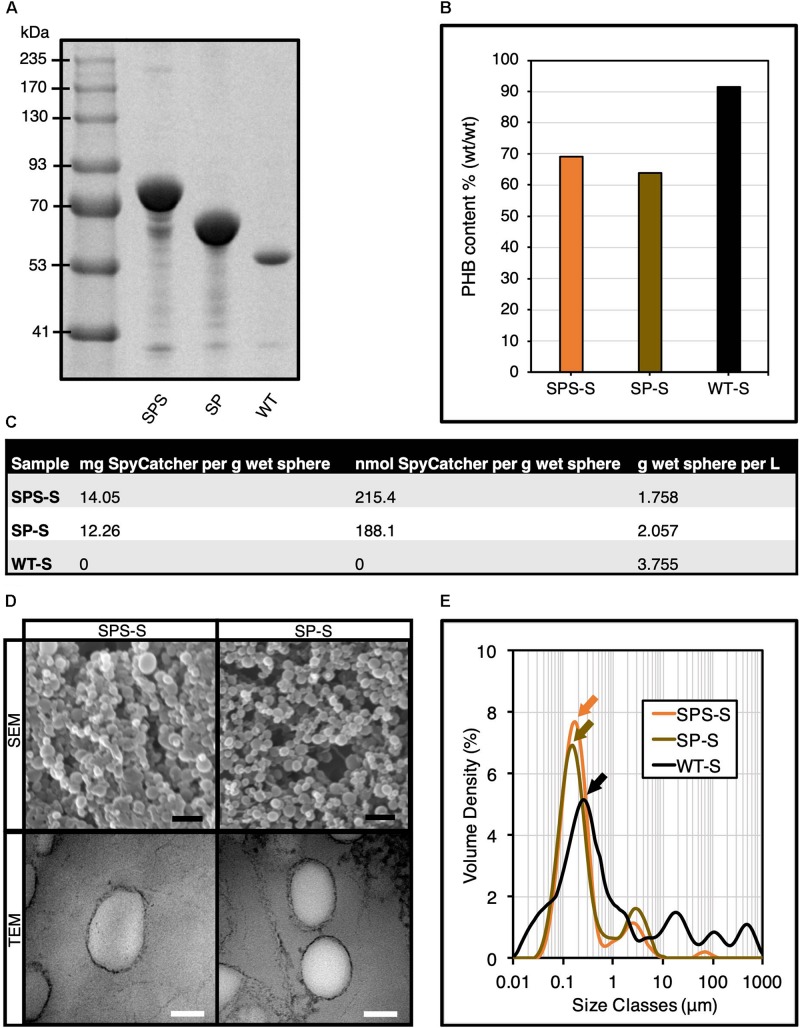
Production and characterization of SpyCatcher-coated PHA spheres. **(A)** SDS-PAGE analysis of various isolated SpyCatcher-coated PHA spheres. **(B)** Compositional analysis of SpyCatcher-coated PHA spheres by GC-MS analysis. **(C)** Production yields of SpyCatcher domains displayed on PHA spheres. **(D)** SEM and TEM micrographs of SpyCatcher-coated PHA spheres. Black scale bar, 1 μm; white scale bar, 100 nm. **(E)** Particle size distribution of SpyCatcher-coated PHA spheres by DLS analysis (mean, *n* = 3). SPS, SpyCatcher-PhaC-SpyCatcher fusion protein; SP, SpyCatcher-PhaC fusion protein; WT, wild-type PhaC; SPS-S, SPS fusion protein-displaying PHA spheres; SP-S, SP fusion protein-displaying PHA spheres; WT-S, WT-displaying PHA spheres.

We used genetically engineered *E. coli* to produce the SpyCatcher-coated PHA spheres, where we utilized isopropyl β-D-1-thiogalactopyranoside (IPTG) inducible plasmid systems with different antibiotic selection markers for single and/or co-production of SpyCatcher-coated PHA spheres and SpyTagged proteins. We have listed all bacterial strains, plasmids, and primers used in this study in Supplementary Tables S1-S3. We also have included the detailed plasmid construction strategies in the Supporting Information (Supplementary Appendix S1). We showed that gene fusion of SpyCatcher to the N- and C-terminus of surface-exposed PhaC, namely SpyCatcher-PhaC-SpyCatcher fusion protein (SPS) (Supplementary Table S4) resulted in overproduction of SpyCatcher domains on the surface of PHA spheres, using our previously developed SpyCatcher-PhaC (SP) fusion protein as reference (Wong and Rehm, 2018). We did not include PhaC-SpyCatcher fusion protein as it had been determined not to be the optimal (Wong and Rehm, 2018). Figure 2A shows the overproduction of two fusion proteins that liquid chromatography-tandem mass spectrometry (LC-MS/MS) (Supplementary Table S5) reveals to be the SPS and SP fusion proteins. The apparent molecular weight of SPS and SP fusion proteins corresponds to the theoretical masses of 81.8 kDa, and 68.4 kDa, respectively, and were greater than only wild-type PhaC (WT) at 55.5 kDa. Unlike SP fusion protein-displaying PHA spheres (SP-S), mixing the SPS fusion protein-displaying PHA spheres (SPS-S) with any SpyTagged proteins will give rise to three different ligated protein products (Supplementary Figure S4).

We also performed compositional analysis of different SpyCatcher-coated PHA spheres, where we compared the PHA composition of SPS-S with our previously developed SP-S (Wong and Rehm, 2018) as reference. We used gas chromatography-mass spectrometry (GC-MS) to determine the PHA composition of our recombinant PHA spheres, using pure poly-(*R*)-3-hydroxybutyrate (PHB) as standard (Supplementary Figure S5). We confirmed the production of different recombinant PHA spheres, where PHB contributed to ∼65-70% of the sphere dry weight, which was significantly lower than WT-displaying PHA spheres (WT-S) (Figure 2B). Lower PHB content aligned with increased fusion protein content (Figure 2B). Hence variation in protein production might contribute to variation in PHB content. Additionally, we quantified the production yields of the PHA spheres (Figure 2C). Scanning electron microscopy (SEM) and transmission electron microscopy (TEM) analyses indicated the successful self-assembly of genetically engineered PHA spheres into the expected spherical shape (Figure 2D). We also performed DLS analysis to determine the particle size and size distribution of our samples (Figure 2E). The particle size distribution obtained from DLS analysis showed monodispersity for both SPS-S and SP-S, with a major peak at ∼176 nm (orange arrow) and ∼155 nm (gold arrow), respectively, which were smaller than WT-S at ∼259 nm (black arrow).

### Surface Functionalization of SpyCatcher-Coated PHA Spheres Using Processes 1-3

We proposed three streamlined processes (processes 1-3) to functionalize the plain SpyCatcher-coated PHA spheres as illustrated in Figure 1B and the detailed flowcharts in Supplementary Figures S1–S3. To visualize the accessibility of the SpyCatcher domains immobilized to PHA spheres available for covalent ligation with the SpyTagged proteins, we first constructed an N-terminally SpyTagged *A. victoria* GFP, namely SpGFP, using the gene fusion approach to enable directed protein ligation to SpyCatcher-coated PHA spheres (Supplementary Table S4). Process 1 describes the *in vivo* modular functionalization of SpyCatcher-coated PHA spheres, where the production of both SpyCatcher-coated PHA spheres and SpyTagged proteins take place within the same cell, resulting *in vivo* functionalization prior isolation of PHA spheres. In process 2, we mix the isolated SpyCatcher-coated PHA spheres with the cleared cell lysate containing soluble SpyTagged proteins to produce *ex vivo* functionalized PHA spheres. In process 3, we implement a cruder version of process 2, where we mix cells containing SpyCatcher-coated PHA spheres and cells containing SpyTagged proteins and then subject them together to cell lysis.

**FIGURE 3:**
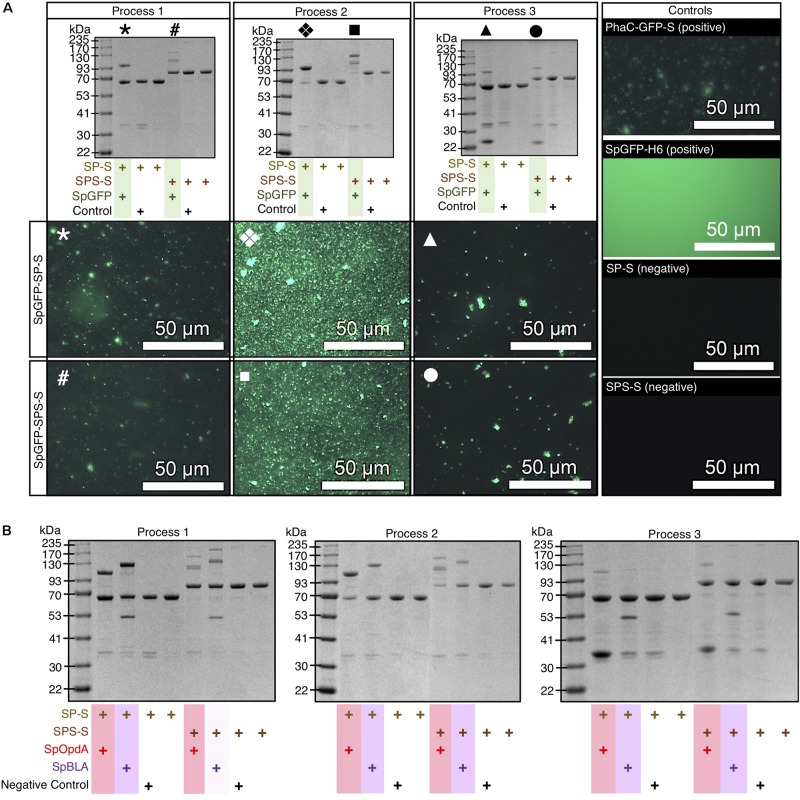
Modular functionalization of SpyCatcher-coated PHA spheres with various SpyTagged proteins implementing processes 1-3 using different plain SpyCatcher-coated PHA spheres as negative controls. **(A)** SDS-PAGE and fluorescence microscopy analyses of SpGFP immobilized on SP-Ss and SPS-Ss. **(B)** SDS-PAGE analysis of SpOpdA and SpBLA immobilized on SP-Ss and SPS-Ss. SP-S, SP fusion protein-displaying PHA spheres; SPS-S, SPS fusion protein-displaying PHA spheres; SpGFP, SpyTagged GFP; SpGFP-H6, SpyTagged GFP bearing His6; SpOpdA, SpyTagged OpdA; SpBLA, SpyTagged BLA; SpGFP-SP-S, SpGFP-SP ligated protein-displaying PHA spheres; SpGFP-SPS-S, SpGFP-SPS ligated protein-displaying PHA spheres; PhaC-GFP-S, PhaC-GFP fusion protein-displaying PHA spheres. *, SpGFP-SP-S produced using process 1; #, SpGFP-SPS-S using process 1; ❖, SpGFP-SP-S produced using process 2; ■, SpGFP-SPS-S produced using process 2; ▲, SpGFP-SP-S produced using process 3; 🌑, SpGFP-SPS-S produced using process 3.

We found that the resulting SpGFP attached to SP-S and SPS-S *in vivo* using process 1 (Figure 3A and Supplementary Figure S6). Additional protein bands appeared at 94.2 kDa, corresponding to SpGFP-SP ligated protein (SpGFP-SP-L), and 116.5 kDa and 140.1 kDa for SpGFP-SPS ligated proteins (SpGFP-SPS-Ls). These protein bands appeared above the molecular weight corresponding to SP (68.4 kDa) and SPS (81.8 kDa) only fusion proteins. At ∼34% and ∼27% surface coverage of SpGFP-SP-L formed on SP-S (SpGFP-SP-S) and SpGFP-SPS-Ls on SPS-S (SpGFP-SPS-S), respectively, we observed bright fluorescence using fluorescence microscopy (Figure 3A). In addition, we quantified the amount of SpGFP-functionalized SPS-S and SP-S produced using process 1 (Supplementary Table S6). Interestingly, we found that direct mixing of isolated SpyCatcher-coated PHA spheres with the SpGFP-containing cleared cell lysate using process 2 resulted in protein band migration of a larger fraction of SP and SPS fusion proteins to SpGFP-SP-L and SpGFP-SPS-Ls, respectively, as revealed by SDS-PAGE analysis (Figure 3A). We also noted that the amount of SpGFP immobilized to both SP-S and SPS-S were ∼3-4 fold higher than those observed in process 1. Consequently, SpGFP-SP-S and SpGFP-SPS-S prepared using process 2 showed a higher fluorescence intensity than those prepared using process 1.

Meanwhile, we noted that SpGFP-SP-S and SpGFP-SPS-S prepared using process 3 was the least efficient approach of all processes, as reflected by the faint protein bands of SpGFP-SP-L and SpGFP-SPS-Ls, at a total surface SpGFP coverage of only ∼11% and ∼17%, respectively, on SpGFP-SP-S and SpGFP-SPS-S (Figure 3A). We also noted an extra protein band at 25.5 kDa, that corresponded to SpGFP as confirmed by LC-MS/MS (Supplementary Table S5), as part of the protein profile of SpGFP-SP-S and SpGFP-SPS-S produced using process 3. We deduced that this could be due to specific non-covalent binding between SpyTag and SpyCatcher, where ligation was not completed. SpGFP was able to diffuse into the interface layer between the bulk phase (cell lysate) and the solid surface of PHA spheres prior to protein ligation. However, the covalent ligation between the SpyTag and SpyCatcher was possibly still incomplete after the cell lysis step, due to the much shorter time for SpGFP to facilitate protein ligation onto the SpyCatcher domains on PHA spheres using process 3, compared to the other processes. Also, a large amount of cellular debris and background proteins might have contributed to the lower protein ligation efficiency. Although, we initially postulated that the presence of this extra band was caused by the incomplete disruption of *E. coli* containing the SpGFP. However, this phenomenon is unlikely as we observed an overall low level of background proteins in plain SPS-S and SP-S preparations using process 3 (Figure 3A). We summarized the amount of SpGFP-functionalized SPS-S and SP-S manufactured using process 3 in Supplementary Table S7. Nevertheless, all the SpGFP-SP-Ss and SpGFP-SPS-Ss, including those of prepared using process 3 could emit green fluorescence similar to those of positive controls, soluble SpyTagged GFP bearing His6 tag (SpGFP-H6) (Wong and Rehm, 2018) and PhaC-GFP fusion protein-displaying PHA spheres (PhaC-GFP-S) (Jahns and Rehm, 2009) prepared using the direct gene fusion method, and in contrast to the negative controls (Figure 3A).

After successful functionalization of SP-S and SPS-S using SpGFP and to demonstrate the versatility of our proposed processes, we designed further SpyTagged proteins representing diverse functions such as two different enzymes. The chosen enzyme candidates were the dimeric organophosphohydrolase (OpdA), an enzyme from *Agrobacterium radiobacter* that can hydrolyze organophosphate pesticides, and the monomeric α-amylase from *Bacillus licheniformis* (BLA), a thermophilic α-linked polysaccharide-degrading enzyme that can hydrolyze starch. We fused SpyTag peptide to the N-terminus of both enzymes, to create SpOpdA and SpBLA (Supplementary Table S4). The SDS-PAGE profiles of all *in vivo* and *ex vivo* enzyme-functionalized SP-S and SPS-S using processes 1-3 are presented in Figure 3B. Briefly, for process 1, the surface coverage of SpyTagged enzymes on SP-S and SPS-S varied from ∼30 to 51% (Figure 3B and Supplementary Table S6). We summarized the amount of functionalized SPS-S and SP-S displaying these tagged enzymes utilizing process 1 in Supplementary Table S6. Interestingly, we noticed a distinct protein band (52.3 kDa) corresponding to SpBLA, confirmed by LC-MS/MS (Supplementary Table S5), as part of the protein profile of both SpBLA-SP ligated protein-displaying PHA spheres (SpBLA-SP-S) and SpBLA-SPS ligated protein-displaying PHA spheres (SpBLA-SPS-S) (Figure 3B and Supplementary Table S6).

We propose that the aforementioned specific binding occurred but with incomplete ligation. Since this phenomenon is unique to SpBLA only using process 1, we suggest that this could be due to its higher molecular weight compared to other SpyTagged proteins. Hence, possibly longer reaction time was necessary to allow complete ligation of SpBLA with the SpyCatcher domains on PHA spheres since larger proteins are more prone to steric hindrance for protein ligation as noted previously (Thrane et al., 2016). Meanwhile, functionalization of SP-S and SPS-S using process 2 achieved up to ∼76% sphere surface coverage using both the SpyTagged enzymes of interest, with distinctive clear protein bands corresponding to the ligated products only (Figure 3B). However, the overall sphere surface coverage of SP-S and SPS-S by both SpyTagged enzymes using process 3 was less than satisfactory, ranging from ∼<1 to 16% for both SP-S and SPS-S (Figure 3B) due to incomplete ligation as noted in the case of SpGFP immobilization. We also further quantified the amount of enzyme-functionalized SPS-S and SP-S yielded using process 3 (Supplementary Table S7).

**FIGURE 4:**
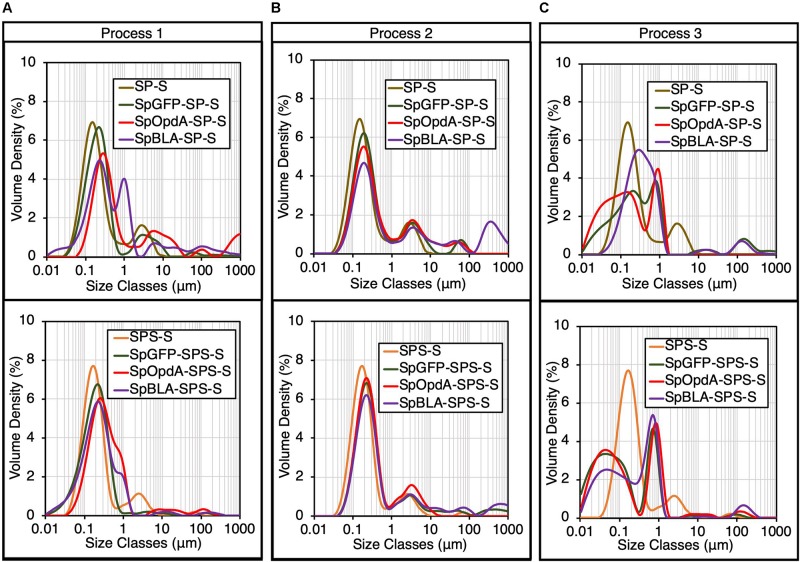
Particle size distribution of various functionalized SpyCatcher-coated PHA spheres produced using processes 1-3 by DLS analysis (mean, *n* = 3). The particle size distribution of plain SP-S and SPS-S determined in Figure 2 are shown as negative controls. Particle size distribution of various functionalized SP-Ss and SPS-Ss produced using **(A)** process 1, **(B)** process 2, or **(C)** process 3. SP-S, SP fusion protein-displaying PHA spheres; SPS-S, SPS fusion protein-displaying PHA spheres; SpGFP-SP-S, SpGFP-SP ligated protein-displaying PHA spheres; SpGFP-SPS-S, SpGFP-SPS ligated protein-displaying PHA spheres; SpOpdA-SP-S, SpOpdA-SP ligated protein-displaying PHA spheres; SpOpdA-SPS-S, SpOpdA-SPS ligated protein-displaying PHA spheres; SpBLA-SP-S, SpBLA-SP ligated protein-displaying PHA spheres; SpBLA-SPS-S, SpBLA-SPS ligated protein-displaying PHA spheres.

Next, all the functionalized SP-Ss and SPS-Ss were subjected to DLS analysis. Particle size distribution analysis revealed that immobilizing SpyTagged proteins onto SP-S and SPS-S using processes 1-2 slightly increased the diameter of individual SP-S and SPS-S (Figures 4A,B). This outcome implies successful ligation of various SpyTagged proteins to the SpyCatcher-coated PHA spheres, without affecting the assembled architecture and monodispersity of SP-S and SPS-S in general. The high polydispersity of SpBLA-SP-S obtained using process 1 indicates the slight potential inconsistency of functionalized PHA spheres using the *in vivo* approach (Figure 4A). We also observed a high degree of particle polydispersity and likely altered architecture of PHA spheres in the case of samples prepared using process 3, possibly due to the excessive mechanical strain on the PHA spheres during the functionalization process (Figure 4C).

### Enzymatic Performance of Functionalized SpyCatcher-Coated PHA Spheres Using the Proposed Processes

**FIGURE 5:**
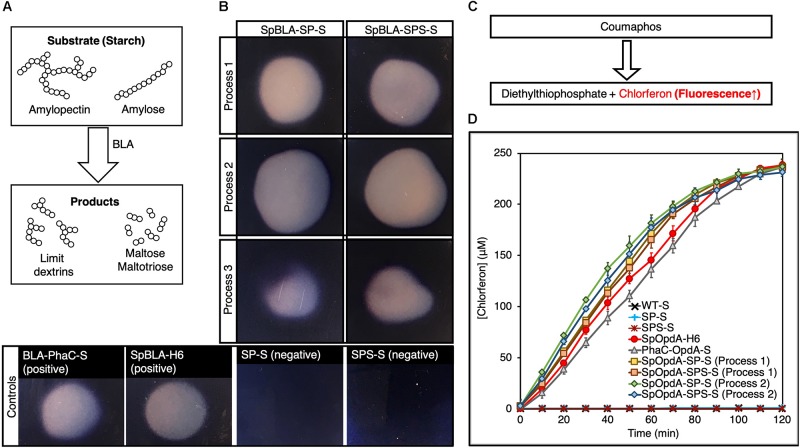
Enzymatic assay of SpBLA and SpOpdA functionalized SpyCatcher-coated PHA spheres. **(A)** Schematic illustration of starch hydrolysis by BLA. **(B)** Formation of clear hydrolytic zone on Lugol’s iodine stained 1% starch agar plate hydrolyzed by SpBLA-SP-S and SpBLA-SPS-S prepared using processes 1-3. **(C)** Schematic of OpdA activity assay using coumaphos as substrate. **(D)** Reaction time course of SpOpdA-SP-S and SpOpdA-SPS-S coumaphos hydrolysis to chlorferon by functionalized spheres produced using processes 1-2, with appropriate controls (mean ± 1 SD, *n* = 3). SP-S, SP fusion protein-displaying PHA spheres; SPS-S, SPS fusion protein-displaying PHA spheres; WT-S, WT-displaying PHA spheres; SpBLA-H6, SpyTagged BLA bearing His6 tag; SpOpdA-H6, SpyTagged OpdA bearing His6 tag; SpBLA-SP-S, SpBLA-SP ligated protein-displaying PHA spheres; SpBLA-SPS-S, SpBLA-SPS ligated protein-displaying PHA spheres; SpOpdA-SP-S, SpOpdA-SP ligated protein-displaying PHA spheres; SpOpdA-SPS-S, SpOpdA-SPS ligated protein-displaying PHA spheres.

After demonstrating successful immobilization of SpyTagged enzymes onto both SP-S and SPS-S, we first qualitatively tested the enzymatic performance of immobilized BLA on 1% (w/v) starch agar (Figure 5A). We used soluble SpyTagged BLA bearing His6 tag (SpBLA-H6) (Wong and Rehm, 2018) and BLA-PhaC fusion protein displayed on PHA spheres (BLA-PhaC-S) (Rasiah and Rehm, 2009) generated using direct gene fusion method as positive controls. All BLA-containing samples created a clear transparent zone on a starch agar plate stained by Lugol’s solution, indicating starch degradation (Figure 5B).

Then, we quantitatively determined the enzymatic performance of both soluble and immobilized forms of OpdA using coumaphos as substrate (Figure 5C), and by assessing the liberated chlorferon from coumaphos degradation relative to a standard curve (Supplementary Figure S7). We performed protein quantification of both soluble and covalently immobilized OpdA (with the relevant controls) using densitometric analysis by SDS-PAGE with a bovine serum albumin (BSA) standard curve (Supplementary Figures S8–S16). All standard curves were linear with *R*^2^ values of at least ∼0.98. For the quantitative OpdA assay, we excluded samples prepared by process 3 due to the extremely low amount of OpdA covalently immobilized to PHA spheres. We also used our previously developed constructs, soluble SpyTagged OpdA bearing His6 tag (SpOpdA-H6) (Wong and Rehm, 2018) and PhaC-OpdA fusion protein displayed on PHA spheres (PhaC-OpdA-S) (Blatchford et al., 2012) prepared using direct gene fusion method as positive controls. We observed subtle improvements in the catalytic activity of immobilized OpdA compared to the positive controls (Figure 5D). The catalytic activity of SpOpdA-SP ligated protein-displaying PHA spheres (SpOpdA-SP-S) and SpOpdA-SPS ligated protein-displaying PHA spheres (SpOpdA-SPS-S) produced by process 2 (5.43 ± 0.3 U/mg and 5.29 ± 0.4 U/mg) and those produced by process 1 (5.14 ± 0.2 U/mg and 4.95 ± 0.4 U/mg) higher than SpOpdA-H6 (4.42 ± 0.4 U/mg) and PhaC-OpdA-S (4.17 ± 0.2 U/mg). This observation is consistent with macromolecular crowding increasing enzyme activity as discussed previously (Wong and Rehm, 2018), as the surface densities of SpOpdA immobilized on individual SP-S and SPS-S using process 2 are higher than that of those produced using process 1. A higher density of OpdA clustering on individual PHA spheres created the excluded volume effect, which in turn drives the coumaphos conversion rate forward.

### Design and Production of Bimodular PHA Spheres

**FIGURE 6:**
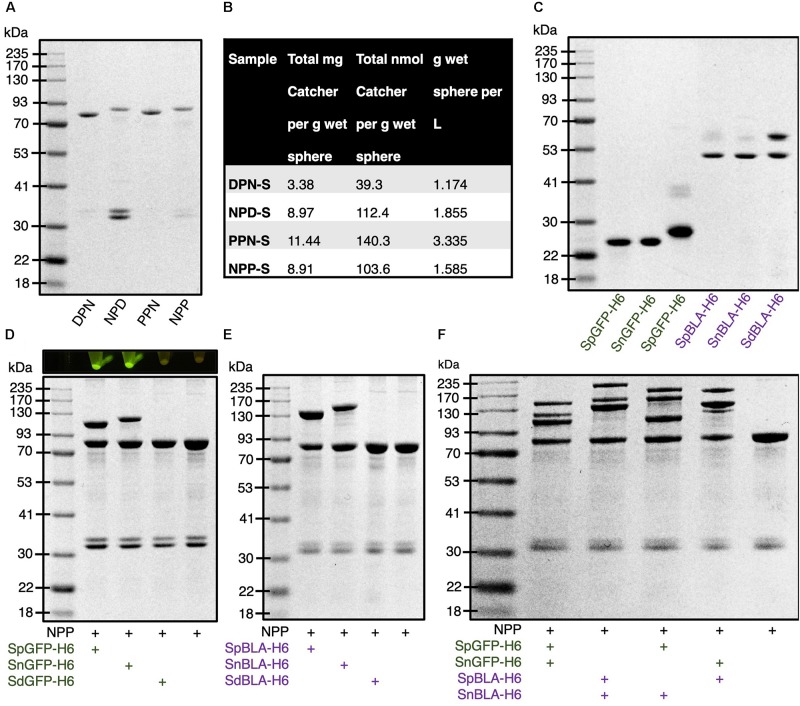
Production and functionalization of various Catcher domain-coated PHA spheres *in vitro*. **(A)** SDS-PAGE analysis of various Catcher domains displayed on PHA spheres. **(B)** Production yields of various Catcher domain-coated on PHA spheres. **(C)** SDS-PAGE analysis of various tagged GFPs and BLAs. **(D)** SDS-PAGE analysis of various tagged GFPs immobilized on NPP-Ss and visualized by blue light exposure. **(E)** SDS-PAGE analysis of various tagged BLAs immobilized on NPP-Ss. **(F)** SDS-PAGE analysis of simultaneous dual-functionalization of NPP-Ss using various tagged GFPs and BLAs. DPN, SdyCatcher-PhaC-SnoopCatcher fusion protein; NPD, SnoopCatcher-PhaC-SdyCatcher fusion protein; PPN, SpyCatcher-PhaC-SnoopCatcher fusion protein; NPP, SnoopCatcher-PhaC-SpyCatcher fusion protein; DPN-S, DPN fusion protein-displaying PHA spheres; NPD-S, NPD fusion protein-displaying PHA spheres; PPN-S, PPN fusion protein-displaying PHA spheres; NPP-S, NPP fusion protein-displaying PHA spheres; SpGFP-H6, SpyTagged GFP bearing His6 tag; SnGFP-H6, SnoopTagged GFP bearing His6 tag; SdGFP-H6, SdyTagged GFP bearing His6 tag; SpBLA-H6, SpyTagged BLA bearing His6 tag; SnBLA-H6, SnoopTagged BLA bearing His6 tag; SdBLA-H6, SdyTagged BLA bearing His6 tag.

To construct bimodular PHA spheres based on the covalent site-specific protein ligation technology, we designed several fusion proteins consisting of combinations of orthogonal Catcher pairs (SpyCatcher, SnoopCatcher, and SdyCatcher_DANG Short_) genetically fused to the N-terminus and C-terminus of PhaC for the purpose of this study. They are SdyCatcher-PhaC-SnoopCatcher fusion protein (DPN), SnoopCatcher-PhaC-SdyCatcher fusion protein (NPD), SpyCatcher-PhaC-SnoopCatcher fusion protein (PPN), and SnoopCatcher-PhaC-SpyCatcher fusion protein (NPP) (Supplementary Table S4), as detailed in the Supporting Information (Supplementary Appendix S1). Then we inserted the constructed genes into IPTG-inducible plasmid vectors and further transformed into recombinant *E. coli* for the biosynthesis of various combinations of Catcher domain-displaying PHA spheres. Supplementary Tables S1-S3 list all the bacterial strains, plasmids, and primers used for this study. Although theoretically possible, the SpyCatcher–SdyCatcher pair was not considered in this study due to the reported low level of cross-reactivity between these two Tag/Catcher pairs in the literature (Tan et al., 2016). Figure 6A illustrates the overproduction of the various fusion proteins displayed on the surface of PHA spheres, providing a high density of Catcher domains spatially distributed on the surface of PHA spheres. The apparent molecular weights of DPN, NPD, PPN, and NPP fusion proteins correspond to the theoretical masses of 79.8 kDa, 86.0 kDa, 81.5 kDa, and 86.3 kDa, respectively. Furthermore, we also tabulated the production yields of DPN fusion protein-displaying PHA spheres (DPN-S), NPD fusion protein-displaying PHA spheres (NPD-S), PPN fusion protein-displaying PHA spheres (PPN-S), and NPP fusion protein-displaying PHA spheres (NPP-S) (Figure 6B).

Next, to allow simultaneous covalent ligation of multiple proteins onto the various Catcher domain-coated PHA spheres *in vitro* in one-step, we incorporated different peptide tags (e.g., SnoopTag and SdyTag) to the N-terminus of both GFP and BLA. These peptide tags are covalently specific to their respective Catcher domains (e.g., SnoopCatcher and SdyCatcher). In addition, to enable simple purification of these fusion proteins using Ni-NTA metal affinity chromatography for *in vitro* protein ligation, we fused a hexahistidine (His6) tag to the C-terminus of these fusion proteins (Supplementary Table S4). This configuration could potentially avoid steric hindrance between the covalent tag and hexahistidine tag (Wong and Rehm, 2018). The genetic fusion of these peptide tags to the selected proteins resulted in the generation of SnoopTagged GFP bearing His6 tag (SnGFP-H6), SnoopTagged BLA bearing His6 tag (SnBLA-H6), SdyTagged GFP bearing His6 tag (SdGFP-H6), and SdyTagged BLA bearing His6 tag (SdBLA-H6) (Supplementary Table S4). Then, we recombinantly biosynthesized these soluble fusion proteins in *E. coli* BL21(DE3) strain. As anticipated, the fusion of these peptide tags did not hinder the recombinant production and Ni-NTA affinity purification of these tagged GFPs and BLAs, using our previously developed SpGFP-H6 and SpBLA-H6 fusion proteins as reference (Figure 6C; Wong and Rehm, 2018).

We performed the densitometry analysis for all the Catcher domain-displaying PHA spheres and purified tagged proteins using a BSA standard curve (Supplementary Figures S17–S19). The concentration of each sample was diluted to fit into the linear range of the standard, where the value of *R*^2^ of the linear curve obtained for each densitometric analysis was at least ∼0.99.

### Screening of Bimodular PHA Spheres Suitable for Efficient Simultaneous Dual-Functionalization

For validation of the accessibility of tagged proteins for covalent protein ligation with various Catcher domain-displaying PHA spheres, we first incubated DPN-S, NPD-S, PPN-S, and NPP-S with excess SpGFP-H6, SnGFP-H6, and SdGFP-H6 *in vitro*. We observed varying levels of protein ligation after incubation of tagged GFPs with various combinations of Catcher domain-displaying PHA spheres (Figure 6D and Supplementary Figures S20–S22). It is suggested that only a small fraction of DPN fusion protein displayed on the PHA sphere can ligate with SpGFP-H6 and SnGFP-H6 as visualized by SDS-PAGE analysis (Supplementary Figure S20). These samples are able to fluoresce under blue light when compared to the control PHA spheres. Note that although SdGFP-DPN ligated proteins (SdGFP-DPN-L) could not be visualized clearly by SDS-PAGE, pelleted SdGFP-DPN-L-displaying PHA spheres (SdGFP-DPN-S) emitted very weak green fluorescence under blue light (Supplementary Figure S20).

Similarly, the covalent tag of both SpGFP-H6 and SdGFP-H6 showed limited accessibility to the Catcher domains displayed on NPD-S individually, where the majority of the NPD fusion proteins did not undergo isopeptide covalent ligation after incubation (Supplementary Figure S21). Interestingly, we noticed that ∼42% of the NPD fusion proteins displayed on PHA spheres were able to ligate with SnGFP-H6, forming SnGFP-NPD ligated protein (SnGFP-NPD-L) with a molecular weight of 111.1 kDa (Supplementary Figure S21). This is supported by the strong fluorescence emitted by SnGFP-NPD-L-displaying PHA spheres (SnGFP-NPD-S) under blue light (Supplementary Figure S21). Meanwhile, ∼45% of PPN fusion protein displayed on PHA spheres was able to immobilize purified SpGFP *in vitro*, as shown on SDS-PAGE indicating a large fraction of PPN fusion proteins forming SpGFP-PPN ligated protein (SpGFP-PPN-L) with a molecular weight of114.1 kDa, much larger when compared to immobilized SdGFP-H6 and SnGFP-H6 on PPN-S (Supplementary Figure S22). This is further evidenced by the higher fluorescence level of SpGFP-PPN-L-displaying PHA spheres (SpGFP-PPN-S) when compared to SdGFP-PPN ligated protein-displaying PHA spheres (SdGFP-PPN-S), and PPN-SnGFP ligated protein-displaying PHA spheres (PPN-SnGFP-S) (Supplementary Figure S22).

Interestingly, NPP-S ligated both SpGFP-H6 and SnGFP-H6 without showing notable cross-reactivity (Figure 6D). We observed the formation of intense NPP-SpGFP ligated protein (NPP-SpGFP-L) and SnGFP-NPP ligated protein (SnGFP-NPP-L) at molecular weights of 108.5 kDa and 118.8 kDa, respectively, as visualized by SDS-PAGE analysis (Figure 6D), indicated successful protein ligation. We further found out that ∼49% and ∼35% of NPP fusion protein could ligate with SpGFP-H6 and SnGFP-H6, and both of the ligated proteins displaying PHA spheres able to fluoresce strongly under blue light (Figure 6D). Besides, we noted that NPP-S showed good reaction orthogonality against SdGFP-H6 (Figure 6D). We also observed a similar trend with the use of differently tagged BLAs to functionalize plain NPP-S, which resulted in the formation of NPP-SpBLA ligated protein (NPP-SpBLA-L) (137.4 kDa) and SnBLA-NPP ligated protein (SnBLA-NPP-L) (148.7 kDa) (Figure 6E). Therefore, and due to the non-optimal accessibility of other Catcher domains displayed on DPN-S, NPD-S, and PPN-S by various tagged GFPs, we selected only the NPP-S construct as our prototype for proof-of-concept simultaneous dual functionalization of bimodular PHA spheres. As expected, NPP-S could immobilize various purified SpyTagged and SnoopTagged proteins simultaneously *in vitro*, as unveiled by the generation of numerous ligated proteins that were larger than the NPP fusion protein (86.3 kDa) (Figure 6F). In addition, we implemented the process 2 mentioned above to demonstrate that NPP-S can be readily functionalized without using purified tagged proteins. NPP-S could react with SpGFP-H6 and SnGFP-H6 in cleared *E. coli* lysate, individually and simultaneously, as revealed by the formation of various ligated proteins that appeared above the molecular weight corresponding to NPP fusion protein (86.3 kDa) (Supplementary Figure S23).

The inconsistent ligation results observed in using DPN-S, NPD-S, and PPN-S as protein immobilization platforms, where we fused various Catcher domains at the different insertion sites of the PHA-binding PhaC, could be due to the misfolding of the fusion proteins displayed on PHA spheres. The individual components of the designed fusion proteins, especially in the case of SnoopCatcher fused to the C-terminus of PhaC in the current study, possibly could not fully replicate the native protein conformation (e.g., non-optimal protein folding) as noted previously due to steric hindrance (Hooks et al., 2013). Failure in fully replicating the native structure of the individual protein domains in the format of fusion protein could result in impaired protein ligation, e.g., restricted accessibility of the tagged proteins to the reactive site of the Catcher domains. In addition, WT -displaying PHA spheres and their recombinant variants often exhibited a negative surface charge at pH 7.5. The isoelectric point (pI) of PHA spheres in suspension typically varies between approximately 5-6 (González-Miro et al., 2017; Gonzalez-Miro et al., 2018a, b). However, the predicted pI of the SdyTag peptide itself is pH 3.9, much lower than those compared to both SpyTag and SnoopTag at pH 8.6 and pH 8.5, respectively (Gasteiger et al., 2005). Therefore, at a ligation reaction pH value of 7.5, both SdyTag peptide and PHA spheres are predominantly negatively charged. Consequently, electrostatic repulsion between these two components is likely responsible for the low ligation yield observed between the SdyTagged proteins onto the PHA spheres.

### Structural Characterization of Selected Bimodular PHA Spheres

**FIGURE 7:**
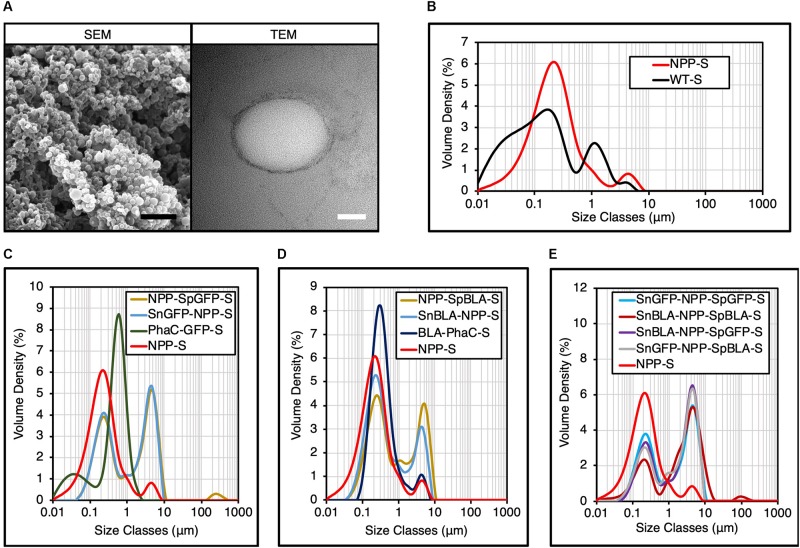
Structural characterization of various NPP-Ss. **(A)** SEM and TEM micrographs of plain NPP-S. Black scale bar, 1 μm; white scale bar, 100 nm. **(B)** Particle size distribution of plain NPP-S by DLS analysis (mean, *n* = 3). **(C)** Particle size distribution of SpGFP-H6 and SnGFP-H6 immobilized NPP-Ss by DLS analysis (mean, *n* = 3) using particle size distribution of plain NPP-S determined in **B** as negative control and PhaC-GFP-S as positive control. **(D)** Particle size distribution of SpBLA-H6 and SnBLA-H6 immobilized NPP-Ss by DLS analysis (mean, *n* = 3) using particle size distribution of plain NPP-S determined in **B** as negative control and BLA-PhaC-S as positive control. **(E)** Particle size distribution of various dual-functionalized NPP-Ss by DLS analysis (mean, *n* = 3) using particle size distribution of plain NPP-S determined in **B** as negative control. NPP-S, NPP fusion protein-displaying PHA spheres; WT-S, WT-displaying PHA spheres; NPP-SpGFP-S, NPP-SpGFP ligated protein-displaying PHA spheres; SnGFP-NPP-S, SnGFP-NPP ligated protein-displaying PHA spheres; PhaC-GFP-S, PhaC-GFP fusion protein-displaying PHA spheres; NPP-SpBLA-S, NPP-SpBLA ligated protein-displaying PHA spheres; SnBLA-NPP-S, SnBLA-NPP ligated protein-displaying PHA spheres; BLA-PhaC-S, BLA-PhaC fusion protein-displaying PHA spheres; SnGFP-NPP-SpGFP-S, SnGFP-NPP-SpGFP ligated protein-displaying PHA spheres; SnBLA-NPP-SpBLA-S, SnBLA-NPP-SpBLA ligated protein-displaying PHA spheres; SnBLA-NPP-SpGFP-S, SnBLA-NPP-SpGFP ligated protein-displaying PHA spheres; SnGFP-NPP-SpBLA-S, SnGFP-NPP-SpBLA ligated protein-displaying PHA spheres.

To characterize the structure of NPP-S, we analyzed the plain NPP-S using both SEM and TEM. Both electron microscopy techniques confirmed the spherical structure of the NPP-S as indicated by the micrographs (Figure 7A), suggesting successful *in vivo* self-assembly of our bimodular PHA spheres as expected. We also further determined the particle size distribution of various NPP-S using DLS analysis (Figures 7B–E). We found that plain NPP-S is homogeneous and has a narrow particle size distribution peaked at ∼214 nm, similar to that of WT-S (Figure 7B). We then measured the particle size distribution of various functionalized NPP-Ss, using NPP-S determined in Figure 7B, PhaC-GFP-S (Jahns and Rehm, 2009), and BLA-PhaC-S (Rasiah and Rehm, 2009) as controls. The diameter of both single and multiple proteins immobilized individual NPP-Ss increased slightly from ∼214 nm to ∼243 nm, suggesting successful immobilization of various tagged proteins on NPP-S (Figures 7C–E). We also noticed that the particle size distribution PhaC-GFP-S and BLA-PhaC-S peaked at ∼597 nm and ∼314 nm, respectively, further validating the impact of using direct protein fusion approach onto the PHA sphere uniformity as mentioned earlier. However, we also observed consistent aggregation behavior of various functionalized NPP-Ss in the ∼4-5 μm diameter range (Figures 7C–E), in contrary to those observed in functionalized SPS-Ss and SP-Ss (Figures 4A–C).

### Robustness of Functionalized Bimodular PHA Spheres

**FIGURE 8:**
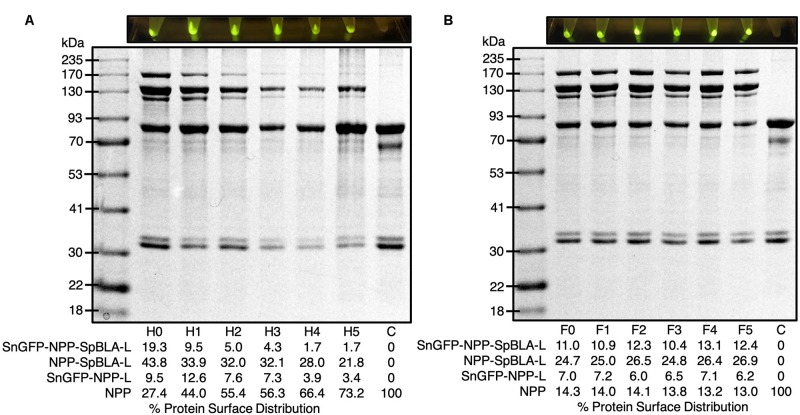
Exposure of plain NPP-S to extreme conditions prior functionalization as visualized by SDS-PAGE analysis and screening of samples under blue light. **(A)** NPP-S could immobilize tagged proteins after five consecutive heat-cooling treatment (H*x*, *x* = round of heat-cooling treatment). **(B)** NPP-S could immobilize tagged proteins after five consecutive freeze-thaw treatment (F*x*, *x* = round of freeze-thaw treatment). NPP, SnoopCatcher-PhaC-SpyCatcher fusion protein; SnGFP-NPP-L, SnGFP-NPP ligated protein; NPP-SpBLA-L, NPP-SpBLA ligated protein; SnGFP-NPP-SpBLA-L, SnGFP-NPP-SpBLA ligated protein.

It is crucial to ensure the robustness of the scaffolding platform against harsh working and storage environments to satisfy different task-specific applications. We subjected the plain NPP-S with up to five rounds of heat-cooling treatment before subsequent functionalization with SnGFP-H6 and SpBLA-H6 *in vitro*. We noted that the NPP-S tend to aggregate vigorously starting on the fourth cycle of heat-cooling treatment visually, making homogenization of the suspension of plain PHA spheres challenging (data not shown). We further found that after five cycles of heat treatment, less than ∼4% of NPP fusion protein on PHA spheres could immobilize SnGFP-H6 to form SnGFP-NPP-L (Figure 8A). Meanwhile, ∼21% of NPP fusion protein on PHA spheres could immobilize SpBLA-H6 to generate NPP-SpBLA-L, and less than ∼2% of NPP fusion protein on PHA spheres could immobilize both SnGFP-H6 and SpBLA-H6 to form SnGFP-NPP-SpBLA ligated proteins (SnGFP-NPP-SpBLA-L) (Figure 8A). With the observation, it is possible to infer that the SnoopCatcher fused to the N-terminus of PhaC is prone to heat denaturation after repeated heat-cooling treatment, and therefore, could not interact with SnGFP-H6 to generate either SnGFP-NPP-L or SnGFP-NPP-SpBLA-L on PHA spheres. After that, we tested the robustness of the NPP-S against multiple freeze-thaw cycles. Plain NPP-Ss were suspended in 50 mM Tris–HCl (pH 7.5) and subjected to up to five cycles of freeze-thaw treatment before incubation under controlled conditions with purified SnGFP-H6 and SpBLA-H6 *in vitro*. We did not include any cryoprotectants in this study to assume the worst-case scenario, e.g., in the event where freezing or thawing of stored samples during storage or transportation. We first confirmed that the resuspension of plain NPP-S using the same buffer still could be performed easily before proceeding to the next step (data not shown). Remarkably, we observed no significant loss of both immobilized proteins on our bimodular PHA spheres after the fifth cycle of freeze-thaw treatment (Figure 8B).

### Functional Performance of Functionalized Bimodular PHA Spheres

**FIGURE 9:**
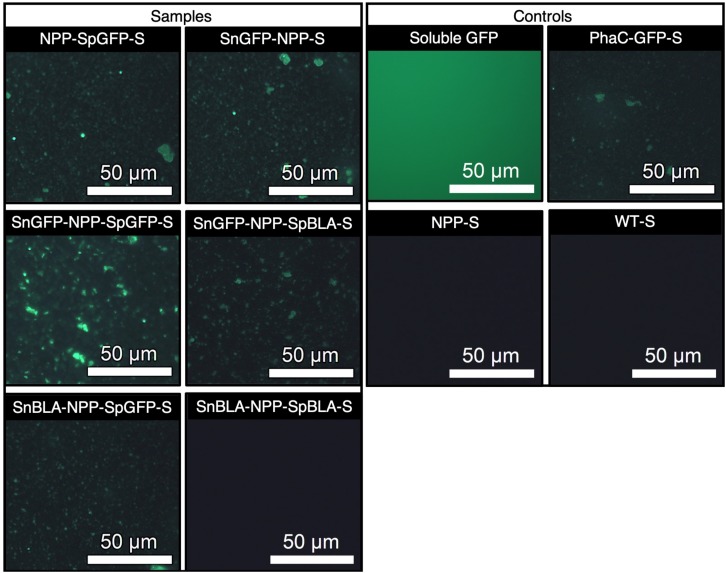
Fluorescence microscopy analysis of SpGFP-H6 and SnGFP-H6 functionalized NPP-Ss with appropriate positive and negative controls. NPP-SpGFP-S, NPP-SpGFP ligated protein-displaying PHA spheres; SnGFP-NPP-S, SnGFP-NPP ligated protein-displaying PHA spheres; SnGFP-NPP-SpGFP-S, SnGFP-NPP-SpGFP ligated protein-displaying PHA spheres; SnGFP-NPP-SpBLA-S, SnGFP-NPP-SpBLA ligated protein-displaying PHA spheres; SnBLA-NPP-SpGFP-S, SnBLA-NPP-SpGFP ligated protein-displaying PHA spheres; SnBLA-NPP-SpBLA-S, SnBLA-NPP-SpBLA ligated protein-displaying PHA spheres; PhaC-GFP-S, PhaC-GFP fusion protein-displaying PHA spheres; NPP-S, NPP fusion protein-displaying PHA spheres; WT-S, wild-type PhaC-displaying PHA spheres.

**FIGURE 10:**
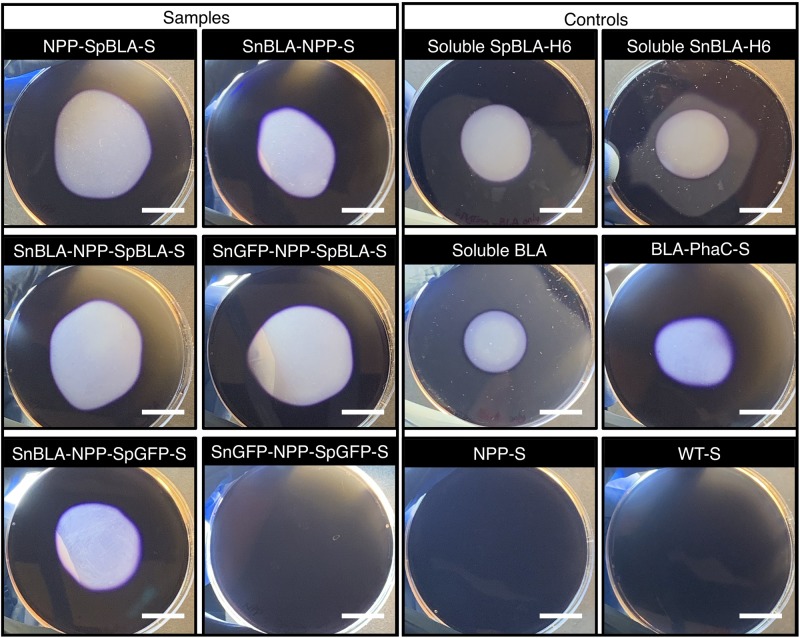
Qualitative starch degradation assay hydrolyzed by SpBLA-H6 and SnBLA-H6 functionalized NPP-Ss with appropriate positive and negative controls. White scale bar, 2 cm. NPP-SpBLA-S, NPP-SpBLA ligated protein-displaying PHA spheres; SnBLA-NPP-S, SnBLA-NPP ligated protein-displaying PHA spheres; SnBLA-NPP-SpBLA-S, SnBLA-NPP-SpBLA ligated protein-displaying PHA spheres; SnGFP-NPP-SpBLA-S, SnGFP-NPP-SpBLA ligated protein-displaying PHA spheres; SnBLA-NPP-SpGFP-S, SnBLA-NPP-SpGFP ligated protein-displaying PHA spheres; SnGFP-NPP-SpGFP-S, SnGFP-NPP-SpGFP ligated protein-displaying PHA spheres; SpBLA-H6, SpyTagged BLA bearing His6 tag; SnBLA-H6, SnoopTagged BLA bearing His6 tag; BLA-PhaC-S, BLA-PhaC fusion protein-displaying PHA spheres; NPP-S, NPP fusion protein-displaying PHA spheres; WT-S, WT-displaying PHA spheres.

To ascertain the functionality of the immobilized proteins on NPP-S, we first carried out fluorescence microscopy analysis on all the functionalized NPP-Ss. We used soluble GFP and PhaC-GFP-S (Jahns and Rehm, 2009; Wong and Rehm, 2018) as positive controls and plain NPP-S and WT-S as negative controls. As expected, all the SpGFP-H6 and SnGFP-H6 immobilized on NPP-Ss in suspension were fluorescent, similar to the positive controls (Figure 9). Meanwhile, non-GFP immobilizing PHA spheres and the negative controls could not emit green fluorescence (Figure 9). We also further tested the functionality of immobilized BLA on NPP-S by loading all the functionalized NPP-Ss on 1% (w/v) starch agar (Wong and Rehm, 2018). We used soluble BLAs and BLA-PhaC-S (Rasiah and Rehm, 2009; Wong and Rehm, 2018) as positive controls and plain NPP-S and WT-S as negative controls. All BLA-immobilized PHA spheres could create a transparent hydrolyzed zone on a Lugol’s iodine stained starch agar plate, comparable to those of positive controls, further suggesting successful starch hydrolysis by the functionalized PHA spheres (Figure 10).

## Discussion

Increasing evidence indicates that fusing different foreign proteins to PHA-associated proteins such as PhaC to facilitate surface functionalization of PHA spheres, in some circumstances could adversely affect the density and functionality of target protein on PHA spheres (Hooks et al., 2013; Hay et al., 2015), as well as the physicochemical uniformity of the recombinant PHA spheres (Rubio-Reyes et al., 2016; Gonzalez-Miro et al., 2018a, b). Therefore, we previously proposed a modular design concept to functionalize PHA spheres using the SpyTag/SpyCatcher chemistry *in vitro* (Wong and Rehm, 2018). This modular design prevents the risk of several inconsistency aspects that are commonly encountered in direct gene fusion recombinant PHA sphere technology (as mentioned) which could hinder its further progress beyond proof-of-concept. However, the use of purified components and subsequent *in vitro* protein ligation could result in higher production costs and time consumption in large-scale manufacturing, and thereby imply inefficiency. Also, the sole reliance of this approach based on SpyTag/SpyCatcher chemistry makes multifunctionalization of the modular PHA spheres less attractive in terms of processability, despite our previous demonstration that different SpyTagged proteins could be spatially distributed on SpyCatcher-coated PHA spheres equally *in vitro* (Wong and Rehm, 2018). We expect that simplified functionalization processes need to be developed in order to overcome the drawbacks of this approach. Therefore, in this study, we sought to implement several cost-effective, innovative strategies in pursuit of simpler modular functionalization of our PHA spheres.

We first explored several simplified processes to functionalize SpyCatcher-coated PHA spheres in order to avoid the necessity of using purified soluble SpyTagged proteins to functionalize our SpyCatcher-coated PHA spheres. Previous studies have reported that similar streamlined processes using SpyTag/SpyCatcher chemistry to functionalize other protein-based nanoparticles (Bruun et al., 2018; Hagen et al., 2018; Zhang et al., 2018a). However, it is of utmost importance to examine these processes in polymeric materials such as PHAs. In general, processes 1-2, but not process 3 could satisfactorily immobilize an adequate amount of SpyTagged proteins to SP-S and SPS-S at varying ligation efficiencies. N-terminally SpyTagged proteins with different quaternary structures and molecular sizes can be immobilized covalently using these processes. Process 1 might be more suitable in the case where efficient processability is paramount for large-scale manufacturing, where functionalized PHA spheres can be isolated directly after bacterial cultivation for immediate usage. We also noted that using process 1, in some instances, could result in non-specific adsorption of one of the SpyTagged proteins onto the surface of PHA spheres (Figure 3B), indicating potential uncertainty in using this process as a general approach to decorate PHA spheres. The cytoplasmic environment within the cell possibly triggered the creation of favorable conditions that facilitated the non-specific adsorption of SpBLA onto the SpyCatcher-coated PHA spheres.

While process 2 requires an extra step when compared to process 1, process 2 could offer a similar level of particle uniformity to that reported previously using the *in vitro* functionalization approach (Wong and Rehm, 2018). This process is still able to avoid the necessity of setting up downstream processes to recover highly purified proteins, which typically account for more than 70% for enzymes, and on some occasions, up to 90% for therapeutic proteins of the recombinant protein production costs (Doran, 2013). These observations indicate that process 2 may be the preferred approach as a standardized process to functionalize PHA spheres in the scenario where stringent control of the particle uniformity and reproducibility are essential. This is especially vital in the case of using PHA spheres in high-value applications, e.g., pharmaceutical and biomedical applications. Inconsistency in the inherent characteristics of particulate scaffolds (e.g., particle size and surface charge) could severely affect the performance and reproducibility of these functionalized scaffolds in some cases (Desai, 2012).

Meanwhile, the second part of this study involves investigating the suitability of the PHA sphere display technology to tolerate the incorporation of two orthogonal reactive Tag/Catcher pairs to achieve multifunctionalization. Successful multifunctionalization of particulate scaffolds, including our previously developed SpyCatcher-coated PHA spheres, have been reported using SpyTag/SpyCatcher chemistry only (Bae et al., 2018; Choi et al., 2018; Wong and Rehm, 2018; Kim et al., 2019). However, these proof-of-concept demonstrations often require careful optimization of the reactant ratio and involve multiple steps to yield precise outcomes, hence are not practical for industrial-scale production. Nevertheless, several recent works also described the utilization of different Tag/Catcher systems to functionalize protein-based scaffolds (Brune et al., 2017; Williams et al., 2018). Therefore, we attempt to design a polymeric PHA scaffold that is able to immobilize different functional proteins simultaneously using various combinations of Tag/Catcher pairs, moving away from sole dependence on the SpyTag/SpyCatcher chemistry.

Initial validation of different Tag/Catcher pairs determined that the performance of constructs other than NPP-S, were less than satisfactory in immobilizing tagged proteins. NPP-S was able to specifically immobilize both purified SnoopTagged and SpyTagged proteins in a simultaneous manner under *in vitro* environments. We also demonstrated the use of process 2 to functionalize NPP-S simultaneously in one-step under *ex vivo* reaction conditions, and thereby suggesting that the processability of this bimodular design is potentially comparable to our previously developed SpyCatcher-coated PHA spheres. However, we noted that the various functionalized NPP-Ss tend to form a high amount of aggregates at ∼4-5 μm observed from the particle size distribution (Figures 7C–E). The undesirable formation of these polymeric clusters could be due to the inherent non-specific interactions independent of the electrostatic interactions, as observed previously (Wong and Rehm, 2018).

Even though direct genetic fusion remains a common approach to functionalize recombinant PHA spheres (Parlane et al., 2016b; Gonzalez-Miro et al., 2019), one of its main limitations is the poor control of immobilized protein density (Wong and Rehm, 2018). We previously introduced the concept of modularity by merging the SpyTag/SpyCatcher system with the PHA sphere technology (Wong and Rehm, 2018). This modular scaffolding platform demonstrated excellent tunability by controlling the ratio of Tag/Catcher (Wong and Rehm, 2018). Although not demonstrated in this study, we expect that our bimodular PHA spheres will provide a similar degree of tunability *in vitro* simultaneously due to the highly specific nature of the Tag/Catcher systems. Ultimately, we hope to expand this unprecedented level of controllability to the *in vivo* and *ex vivo* reaction conditions using processes 1-2. It is feasible to achieve the desired Tag/Catcher reactant ratio avoiding laborious purification steps for *in vivo* and *ex vivo* ligation reactions, by precisely optimizing the production levels of both tagged proteins and Catcher domain-coated PHA spheres. Future studies aiming to enhance this Tag/Catcher PHA sphere technology will include regulated gene expression, different production host and manufacturing conditions (Goverdhana et al., 2005; Gutiérrez-González et al., 2019; Trösemeier et al., 2019).

The findings in this study further elaborate on the information required to develop our modular PHA sphere display technology as an emerging platform for surface display of proteins without compromising its advantages. We envision that these strategies may open new avenues for functionalizing PHA spheres for the cost-effective production of various high-value-added PHA spheres processes. These processes could make the modular functionalization approach more appealing from a cost-effective standpoint (e.g., one-step manufacturing) while able to offer improved particle uniformity compared to the PhaC-based direct gene fusion approach. These positive outcomes further indicate that a versatile toolbox for the robust production of designer PHA spheres could be established.

## Data Availability Statement

All datasets generated for this study are included in the article/Supplementary Material.

## Author Contributions

JW and BR conceived the main conceptual ideas of this study, and designed the study. JW performed all the experiments except part of the DNA cloning work, and took the lead in writing the manuscript in consultation with BR, AS-S, and MG-M. MG-M performed part of the cloning work. BR and AS-S provided the technical feedback. All authors gave approval to the final version of the manuscript.

## Conflict of Interest

BR is co-founder and shareholder of PolyBatics Ltd. that commercializes veterinary TB diagnostic products related to the polyester sphere technology. The remaining authors declare that the research was conducted in the absence of any commercial or financial relationships that could be construed as a potential conflict of interest.
