# Supplementary material for: Covalent Functionalization of Bioengineered Polyhydroxyalkanoate Spheres Directed by Specific Protein-Protein Interactions
Source: Front Bioeng Biotechnol. 2020 Feb 6;8:44. doi: 10.3389/fbioe.2020.00044 (PMC7015861; doi:10.3389/fbioe.2020.00044)
Supplement: Supplementary file 1 [file Table_1.DOCX]

**Table S1.** Bacterial strains used in the current study.

| Bacterial strains | Characteristics | References |
| --- | --- | --- |
| *Escherichia coli* XL1-Blue | *recA1 endA1 gyrA96 thi-1 hsdR17 supE44 RelA1 lac* [F’ *proAB lacI^q^*Z∆*M15* Tn*10 (Tet^r^)]* | Stratagene |
| *Escherichia coli* BL21(DE3) | F^-^*dcm ompT hsdS*(rB^-^mB^-^) gal λ(DE3) | Invitrogen |

**Table S2.** Plasmids constructed and used in the current study.

| Plasmids | Characteristics | References |
| --- | --- | --- |
| pET14b | Ap^r^; T7 promoter. | Novagen |
| pMSC69 | Cm^r^; pBBR1MCS derivative containing genes *phaA* and *phaB* from *C. necator* co-linear to *lac* promoter. | (Amara and Rehm, 2003) |
| pET14b_SpyCatcher_PhaC | pET14b encoding *SpyCatcher* fused to the N-terminus of *phaC* via a linker sequence. | (Wong and Rehm, 2018) |
| pET14b_SpyCatcher_PhaC_  linker_SpyCatcher | pET14b encoding two *SpyCatcher* flanking at both N- and C- termini of *phaC*. | (Wong and Rehm, 2018)* |
| pCOLADuet-1 | Km^r^; T7 promoter; ColA replicon. | Novagen |
| pBluescript_II_SK(+)_SdyCatcher-SnoopCatcher | Ap^r^. | Biomatik |
| pET14b_SpyTag-GFP-His6 | pET14b derivative encoding *SpyTag* at N-terminus of *gfp* and hexahistidine tag at 3’ end of *gfp*. | (Wong and Rehm, 2018) |
| pET14b_SpyTag-OpdA-His6. | pET14b derivative encoding *SpyTag* at N-terminus of *opda* and hexahistidine tag at 3’ end of *opda*. | (Wong and Rehm, 2018) |
| pET14b_SpyTag-BLA-His6. | pET14b encoding *SpyTag* at N-terminus of *bla* and hexahistidine tag at C-terminus of *bla*. | (Wong and Rehm, 2018) |
| pCOLASolo-1_SpyTag-GFP | pCOLADuet-1 derivative encoding *SpyTag* fused at the N-terminus of *gfp* for single protein production. | This study |
| pCOLASolo-1_SpyTag-OpdA | pCOLADuet-1 derivative *SpyTag* fused at the N-terminus of *opda* for single protein production. | This study |
| pCOLASolo-1_SpyTag-BLA | pCOLADuet-1 derivative *SpyTag* fused at the N-terminus of *bla* for single protein production. | This study |
| pET14b_C2 | pET14b derivative consisting *C2 gene*. | Unpublished work |
| pET14b_SdyCatcher-PhaC-SnoopCatcher | pET14b derivative consisting *SdyCatcher* fused to the N-terminus of *phaC* and *SnoopCatcher* to the C-terminus of *phaC* via a linker sequence. | This study |
| pET14b_SnoopCatcher-PhaC-SdyCatcher | pET14b derivative consisting *SnoopCatcher* fused to the N-terminus of *phaC* and *SdyCatcher* to the C-terminus of *phaC* via a linker sequence. | This study |
| pET14b_SpyCatcher-PhaC-SnoopCatcher | pET14b derivative consisting *SpyCatcher* fused to the N-terminus of *phaC* and *SnoopCatcher* to the C-terminus of *phaC* via a linker sequence. | This study |
| pET14b_SnoopCatcher-PhaC-SpyCatcher | pET14b derivative consisting *SnoopCatcher* fused to the N-terminus of *phaC* and *SpyCatcher* to the C-terminus of *phaC* via a linker sequence. | This study |
| pET14b_SnoopTag-L-GFP-His6 | pET14b_PhaC_linker_GFP derivative consisting *SnoopTag* at N-terminus of *gfp* and hexahistidine tag at C-terminus of *gfp*. | This study |
| pET14b_SnoopTag-L-BLA-His6. | pET14b_BLAphaC derivative consisting *SnoopTag* at N-terminus of *bla* and hexahistidine tag at C- terminus of *bla*. | This study |
| pET14b_SdyTag-L-GFP-His6 | pET14b_PhaC_linker_GFP derivative consisting *SdyTag* at N-terminus of *gfp* and hexahistidine tag at C-terminus of *gfp*. | This study |
| pET14b_SdyTag-L-BLA-His6. | pET14b_ BLAphaC derivative consisting *SdyTag* at N-terminus of *bla* and hexahistidine tag at C- terminus of *bla*. | This study |

*constructed previously (Wong and Rehm, 2018) as an intermediate plasmid for construction of pET14b_SpyCatcher_PhaC.

**Table S3.** Primers constructed and used in the current study.

| Primers | Restriction sites | Sequence | References |
| --- | --- | --- | --- |
| NcoI-XhoI-SpyTag | NcoI & XhoI | 5’ATATTTCCATGGGACTCGAGGCTCATATTGTGATGGTGGATGCG | This study |
| AvrII-STOP-OpdA | AvrII | 5’ATATTTCCTAGGTTACGACGCCCGCACGG | This study |
| AvrII-STOP-BLA | AvrII | 5’ATATTTCCTAGGTTAGCGCTGGACGTAGATGGAAACAG | This study |
| AvrII-STOP-GFP | AvrII | 5’ATATTTCCTAGGTTATTTGTATAGTTCATCCATGCCATGTGTAATCCCAG | This study |
| SpeI-(START)-SnoopCatcher | SpeI | 5’ATATATACTAGTATGCATATGAAACCGCTGCGTGGC | This study |
| AvrII-SnoopCatcher | AvrII | 5’ATATATCCTAGGTTTCGGCGGAATCGGTTCATTGG | This study |
| XhoI-SnoopCatcher | XhoI | 5’ATATATCTCGAGCATATGAAACCGCTGCGTGGC | This study |
| BamHI-(STOP)-SnoopCatcher | BamHI | 5’ATATATGGATCCTCATTTCGGCGGAATCGGTTCATTGG | This study |
| SpeI-(START)-SdyCatcher | SpeI | 5’ATATATACTAGTATGGGTAGTAGTGGTCTGAGC | This study |
| AvrII-SdyCatcher | AvrII | 5’ATATATCCTAGGGCTATCCACCCAAATCTGGC | This study |
| XhoI-SdyCatcher | XhoI | 5’ATATATCTCGAGGGTAGTAGTGGTCTGAGC | This study |
| BamHI-(STOP)-SdyCatcher | BamHI | 5’ATATATGGATCCTCAGCTATCCACCCAAATCTGGC | This study |
| SpeI-START-SnoopTag-L-GFP | SpeI | 5’TATACTAGTATGGGGAAACTCGGCGATATTGAATTTATTAAAGTGAACAAAGGCAGTGGTTCGGGATCAGGAAGTAAAGGAGAAGAACTTTTCACTGGAG | This study |
| **SpyTag-GFP-His6_RVR** | BamHI | 5’ATATTTGGATCCTCAGTGATGATGGTGATGATGTTTGTATAGTTCATCCATGCCATGTGT | (Wong and Rehm, 2018) |
| SpeI-START-SnoopTag-L-BLA | SpeI | 5’TATACTAGTATGGGGAAACTGGGCGATATTGAATTTATTAAAGTGAACAAAGGCAGTGGTTCGGGATCAGGAGCTAACCTGAACGGTACCCTGATG | This study |
| **SpyTag-BLA-His6_RVR** | BamHI | 5’ATATTTGGATCCTCAGTGATGATGGTGATGATGGCGCTGGACGTAGATGGAAACAGA | (Wong and Rehm, 2018) |
| SpeI-START-SdyTag-L-GFP | SpeI | 5’AATACTAGTATGGATCCGATTGTGATGATTGATAACGATAAACCGATTACCGGCAGTGGTTCGGGATCCGGAAGTAAAGGAGAAGAACTTTTCACTGGAG | This study |
| XhoI-STOP-His6-GFP | XhoI | 5’ATATTTCTCGAGTCAGTGATGATGGTGATGATGTTTGTATAGTTCATCCATGCCATGTGT | This study |
| SpeI-START-SdyTag-L-BLA | SpeI | 5’TATACTAGTATGGATCCGATTGTGATGATTGATAACGATAAACCGATTACCGGCAGTGGTTCGGGATCTGGAGCTAACCTGAACGGTACCCTGATG | This study |
| XhoI-STOP-His6-BLA | XhoI | 5’ATATTTCTCGAGTCAGTGATGATGGTGATGATGGCGCTGGACGTAGATGGAAACAGA | This study |

| **Table S4.** Amino acid sequence of fusion proteins | |
| --- | --- |
| Fusion protein | Amino acid sequence |
| Wild-type *Cupriavidus necator* PhaC (WT) | ATGKGAAASTQEGKSQPFKVTPGPFDPATWLEWSRQWQGTEGNGHAAASGIPGLDALAGVKIAPAQLGDIQQRYMKDFSALWQAMAEGKAEATGPLHDRRFAGDAWRTNLPYRFAAAFYLLNARALTELADAVEADAKTRQRIRFAISQWVDAMSPANFLATNPEAQRLLIESGGESLRAGVRNMMEDLTRGKISQTDESAFEVGRNVAVTEGAVVFENEYFQLLQYKPLTDKVHARPLLMVPPCINKYYILDLQPESSLVRHVVEQGHTVFLVSWRNPDASMAGSTWDDYIEHAAIRAIEVARDISGQDKINVLGFCVGGTIVSTALAVLAARGEHPAASVTLLTTLLDFADTGILDVFVDEGHVQLREATLGGGAGAPCALLRGLELANTFSFLRPNDLVWNYVVDNYLKGNTPVPFDLLFWNGDATNLPGPWYCWYLRHTYLQNELKVPGKLTVCGVPVDLASIDVPTYIYGSREDHIVPWTAAYASTALLANKLRFVLGASGHIAGVINPPAKNKRSHWTNDALPESPQQWLAGAIEHHGSWWPDWTAWLAGQAGAKRAAPANYGNARYRAIEPAPGRYVKAKAHMVLAVAIDKR* |
| **SpyCatcher-**PhaC**-SpyCatcher** (**S**P**S**) | M**GAMVDTLSGLSSEQGQSGDMTIEEDSATHIKFSKRDEDGKELAGATMELRDSSGKTISTWISDGQVKDFYLYPGKYTFVETAAPDGYEVATAITFTVNEQGQVTVNGKATKGDAHI**PRHMATGKGAAASTQEGKSQPFKVTPGPFDPATWLEWSRQWQGTEGNGHAAASGIPGLDALAGVKIAPAQLGDIQQRYMKDFSALWQAMAEGKAEATGPLHDRRFAGDAWRTNLPYRFAAAFYLLNARALTELADAVEADAKTRQRIRFAISQWVDAMSPANFLATNPEAQRLLIESGGESLRAGVRNMMEDLTRGKISQTDESAFEVGRNVAVTEGAVVFENEYFQLLQYKPLTDKVHARPLLMVPPCINKYYILDLQPESSLVRHVVEQGHTVFLVSWRNPDASMAGSTWDDYIEHAAIRAIEVARDISGQDKINVLGFCVGGTIVSTALAVLAARGEHPAASVTLLTTLLDFADTGILDVFVDEGHVQLREATLGGGAGAPCALLRGLELANTFSFLRPNDLVWNYVVDNYLKGNTPVPFDLLFWNGDATNLPGPWYCWYLRHTYLQNELKVPGKLTVCGVPVDLASIDVPTYIYGSREDHIVPWTAAYASTALLANKLRFVLGASGHIAGVINPPAKNKRSHWTNDALPESPQQWLAGAIEHHGSWWPDWTAWLAGQAGAKRAAPANYGNARYRAIEPAPGRYVKAKAHMVLAVAIDKRGGGGGLE**GAMVDTLSGLSSEQGQSGDMTIEEDSATHIKFSKRDEDGKELAGATMELRDSSGKTISTWISDGQVKDFYLYPGKYTFVETAAPDGYEVATAITFTVNEQGQVTVNGKATKGDAHI*** |
| **SpyCatcher-**PhaC (**S**P) | M**GAMVDTLSGLSSEQGQSGDMTIEEDSATHIKFSKRDEDGKELAGATMELRDSSGKTISTWISDGQVKDFYLYPGKYTFVETAAPDGYEVATAITFTVNEQGQVTVNGKATKGDAHI**PRHMATGKGAAASTQEGKSQPFKVTPGPFDPATWLEWSRQWQGTEGNGHAAASGIPGLDALAGVKIAPAQLGDIQQRYMKDFSALWQAMAEGKAEATGPLHDRRFAGDAWRTNLPYRFAAAFYLLNARALTELADAVEADAKTRQRIRFAISQWVDAMSPANFLATNPEAQRLLIESGGESLRAGVRNMMEDLTRGKISQTDESAFEVGRNVAVTEGAVVFENEYFQLLQYKPLTDKVHARPLLMVPPCINKYYILDLQPESSLVRHVVEQGHTVFLVSWRNPDASMAGSTWDDYIEHAAIRAIEVARDISGQDKINVLGFCVGGTIVSTALAVLAARGEHPAASVTLLTTLLDFADTGILDVFVDEGHVQLREATLGGGAGAPCALLRGLELANTFSFLRPNDLVWNYVVDNYLKGNTPVPFDLLFWNGDATNLPGPWYCWYLRHTYLQNELKVPGKLTVCGVPVDLASIDVPTYIYGSREDHIVPWTAAYASTALLANKLRFVLGASGHIAGVINPPAKNKRSHWTNDALPESPQQWLAGAIEHHGSWWPDWTAWLAGQAGAKRAAPANYGNARYRAIEPAPGRYVKAKAHMVRIRLLTKPERKLSWLLPPLSNN***** |
| **SdyCatcher**-PhaC-**SnoopCatcher**  (**D**P**N**) | M**GSSGLSGETGQSGNTTIEEDSTTHVKFSKRDANGKELAGAMIELRNLSGQTIQSWISDGTVKVFYLMPGTYQFVETAAPEGYELAAPITFTIDEKGQIWVDS**PRHMATGKGAAASTQEGKSQPFKVTPGPFDPATWLEWSRQWQGTEGNGHAAASGIPGLDALAGVKIAPAQLGDIQQRYMKDFSALWQAMAEGKAEATGPLHDRRFAGDAWRTNLPYRFAAAFYLLNARALTELADAVEADAKTRQRIRFAISQWVDAMSPANFLATNPEAQRLLIESGGESLRAGVRNMMEDLTRGKISQTDESAFEVGRNVAVTEGAVVFENEYFQLLQYKPLTDKVHARPLLMVPPCINKYYILDLQPESSLVRHVVEQGHTVFLVSWRNPDASMAGSTWDDYIEHAAIRAIEVARDISGQDKINVLGFCVGGTIVSTALAVLAARGEHPAASVTLLTTLLDFADTGILDVFVDEGHVQLREATLGGGAGAPCALLRGLELANTFSFLRPNDLVWNYVVDNYLKGNTPVPFDLLFWNGDATNLPGPWYCWYLRHTYLQNELKVPGKLTVCGVPVDLASIDVPTYIYGSREDHIVPWTAAYASTALLANKLRFVLGASGHIAGVINPPAKNKRSHWTNDALPESPQQWLAGAIEHHGSWWPDWTAWLAGQAGAKRAAPANYGNARYRAIEPAPGRYVKAKAHMVLAVAIDKRGGGGGLE**HMKPLRGAVFSLQKQHPDYPDIYGAIDQNGTYQNVRTGEDGKLTFKNLSDGKYRLFENSEPAGYKPVQNKPIVAFQIVNGEVRDVTSIVPQDIPATYEFTNGKHYITNEPIPPK*** |
| **SnoopCatcher**-PhaC-**SdyCatcher**  **(N**P**D)** | M**HMKPLRGAVFSLQKQHPDYPDIYGAIDQNGTYQNVRTGEDGKLTFKNLSDGKYRLFENSEPAGYKPVQNKPIVAFQIVNGEVRDVTSIVPQDIPATYEFTNGKHYITNEPIPPK**PRHMATGKGAAASTQEGKSQPFKVTPGPFDPATWLEWSRQWQGTEGNGHAAASGIPGLDALAGVKIAPAQLGDIQQRYMKDFSALWQAMAEGKAEATGPLHDRRFAGDAWRTNLPYRFAAAFYLLNARALTELADAVEADAKTRQRIRFAISQWVDAMSPANFLATNPEAQRLLIESGGESLRAGVRNMMEDLTRGKISQTDESAFEVGRNVAVTEGAVVFENEYFQLLQYKPLTDKVHARPLLMVPPCINKYYILDLQPESSLVRHVVEQGHTVFLVSWRNPDASMAGSTWDDYIEHAAIRAIEVARDISGQDKINVLGFCVGGTIVSTALAVLAARGEHPAASVTLLTTLLDFADTGILDVFVDEGHVQLREATLGGGAGAPCALLRGLELANTFSFLRPNDLVWNYVVDNYLKGNTPVPFDLLFWNGDATNLPGPWYCWYLRHTYLQNELKVPGKLTVCGVPVDLASIDVPTYIYGSREDHIVPWTAAYASTALLANKLRFVLGASGHIAGVINPPAKNKRSHWTNDALPESPQQWLAGAIEHHGSWWPDWTAWLAGQAGAKRAAPANYGNARYRAIEPAPGRYVKAKAHMVLAVAIDKRGGGGGLE**GSSGLSGETGQSGNTTIEEDSTTHVKFSKRDANGKELAGAMIELRNLSGQTIQSWISDGTVKVFYLMPGTYQFVETAAPEGYELAAPITFTIDEKGQIWVDS*** |
| **SpyCatcher**-PhaC-**SnoopCatcher** (**P**P**N**) | M**GAMVDTLSGLSSEQGQSGDMTIEEDSATHIKFSKRDEDGKELAGATMELRDSSGKTISTWISDGQVKDFYLYPGKYTFVETAAPDGYEVATAITFTVNEQGQVTVNGKATKGDAHI**PRHMATGKGAAASTQEGKSQPFKVTPGPFDPATWLEWSRQWQGTEGNGHAAASGIPGLDALAGVKIAPAQLGDIQQRYMKDFSALWQAMAEGKAEATGPLHDRRFAGDAWRTNLPYRFAAAFYLLNARALTELADAVEADAKTRQRIRFAISQWVDAMSPANFLATNPEAQRLLIESGGESLRAGVRNMMEDLTRGKISQTDESAFEVGRNVAVTEGAVVFENEYFQLLQYKPLTDKVHARPLLMVPPCINKYYILDLQPESSLVRHVVEQGHTVFLVSWRNPDASMAGSTWDDYIEHAAIRAIEVARDISGQDKINVLGFCVGGTIVSTALAVLAARGEHPAASVTLLTTLLDFADTGILDVFVDEGHVQLREATLGGGAGAPCALLRGLELANTFSFLRPNDLVWNYVVDNYLKGNTPVPFDLLFWNGDATNLPGPWYCWYLRHTYLQNELKVPGKLTVCGVPVDLASIDVPTYIYGSREDHIVPWTAAYASTALLANKLRFVLGASGHIAGVINPPAKNKRSHWTNDALPESPQQWLAGAIEHHGSWWPDWTAWLAGQAGAKRAAPANYGNARYRAIEPAPGRYVKAKAHMVLAVAIDKRGGGGGLE**HMKPLRGAVFSLQKQHPDYPDIYGAIDQNGTYQNVRTGEDGKLTFKNLSDGKYRLFENSEPAGYKPVQNKPIVAFQIVNGEVRDVTSIVPQDIPATYEFTNGKHYITNEPIPPK*** |
| **SnoopCatcher**-PhaC-**SpyCatcher**  (**N**P**P**) | M**HMKPLRGAVFSLQKQHPDYPDIYGAIDQNGTYQNVRTGEDGKLTFKNLSDGKYRLFENSEPAGYKPVQNKPIVAFQIVNGEVRDVTSIVPQDIPATYEFTNGKHYITNEPIPPK**PRHMATGKGAAASTQEGKSQPFKVTPGPFDPATWLEWSRQWQGTEGNGHAAASGIPGLDALAGVKIAPAQLGDIQQRYMKDFSALWQAMAEGKAEATGPLHDRRFAGDAWRTNLPYRFAAAFYLLNARALTELADAVEADAKTRQRIRFAISQWVDAMSPANFLATNPEAQRLLIESGGESLRAGVRNMMEDLTRGKISQTDESAFEVGRNVAVTEGAVVFENEYFQLLQYKPLTDKVHARPLLMVPPCINKYYILDLQPESSLVRHVVEQGHTVFLVSWRNPDASMAGSTWDDYIEHAAIRAIEVARDISGQDKINVLGFCVGGTIVSTALAVLAARGEHPAASVTLLTTLLDFADTGILDVFVDEGHVQLREATLGGGAGAPCALLRGLELANTFSFLRPNDLVWNYVVDNYLKGNTPVPFDLLFWNGDATNLPGPWYCWYLRHTYLQNELKVPGKLTVCGVPVDLASIDVPTYIYGSREDHIVPWTAAYASTALLANKLRFVLGASGHIAGVINPPAKNKRSHWTNDALPESPQQWLAGAIEHHGSWWPDWTAWLAGQAGAKRAAPANYGNARYRAIEPAPGRYVKAKAHMVLAVAIDKRGGGGGLE**GAMVDTLSGLSSEQGQSGDMTIEEDSATHIKFSKRDEDGKELAGATMELRDSSGKTISTWISDGQVKDFYLYPGKYTFVETAAPDGYEVATAITFTVNEQGQVTVNGKATKGDAHI*** |
| **SpyTagged** *Aequorea victoria* green fluorescent protein **(Sp**GFP) | M**AHIVMVDAYKPTK**GGGSKGEELFTGVVPILVELDGDVNGHKFSVSGEGEGDATYGKLTLKFICTTGKLPVPWPTLVTTLTYGVQCFSRYPDHMKRHDFFKSAMPEGYVQERTIFFKDDGNYKTRAEVKFEGDTLVNRIELKGIDFKEDGNILGHKLEYNYNSHNVYIMADKQKNGIKVNFKIRHNIEDGSVQLADHYQQNTPIGDGPVLLPDNHYLSTQSALSKDPNEKRDHMVLLEFVTAAGITHGMDELYK* |
| **SpyTagged Aequorea** *victoria* green fluorescent protein bearing His6 tag **(Sp**GFP-H6) | M**AHIVMVDAYKPTK**GGGSKGEELFTGVVPILVELDGDVNGHKFSVSGEGEGDATYGKLTLKFICTTGKLPVPWPTLVTTLTYGVQCFSRYPDHMKRHDFFKSAMPEGYVQERTIFFKDDGNYKTRAEVKFEGDTLVNRIELKGIDFKEDGNILGHKLEYNYNSHNVYIMADKQKNGIKVNFKIRHNIEDGSVQLADHYQQNTPIGDGPVLLPDNHYLSTQSALSKDPNEKRDHMVLLEFVTAAGITHGMDELYKHHHHHH* |
| **SpyTagged**  *Agrobacterium radiobacter* organophosphohydrolase (**Sp**OpdA) | MGLE**AHIVMVDAYKPTK**GGGSMARPIGTGDLINTVRGPIPVSEAGFTLTHEHICGSSAGFLRAWPEFFGSRKALAEKAVRGLRHARAAGVQTIVDVSTFDIGRDVRLLAEVSRAADVHIVAATGLWFDPPLSMRMRSVEELTQFFLREIQHGIEDTGIRAGIIKVATTGKATPFQELVLKAAARASLATGVPVTTHTSASQRDGEQQAAIFESEGLSPSRVCIGHSDDTDDLSYLTGLAARGYLVGLDRMPYSAIGLEGNASALALFGTRSWQTRALLIKALIDRGYKDRILVSHDWLFGFSSYVTNIMDVMDRINPDGMAFVPLRVIPFLREKGVPPETLAGVTVANPARFLSPTVRAS***** |
| **SpyTagged**  *Agrobacterium radiobacter* organophosphohydrolase bearing His6 tag (**Sp**OpdA-H6) | MGLE**AHIVMVDAYKPTK**GGGSMARPIGTGDLINTVRGPIPVSEAGFTLTHEHICGSSAGFLRAWPEFFGSRKALAEKAVRGLRHARAAGVQTIVDVSTFDIGRDVRLLAEVSRAADVHIVAATGLWFDPPLSMRMRSVEELTQFFLREIQHGIEDTGIRAGIIKVATTGKATPFQELVLKAAARASLATGVPVTTHTSASQRDGEQQAAIFESEGLSPSRVCIGHSDDTDDLSYLTGLAARGYLVGLDRMPYSAIGLEGNASALALFGTRSWQTRALLIKALIDRGYKDRILVSHDWLFGFSSYVTNIMDVMDRINPDGMAFVPLRVIPFLREKGVPPETLAGVTVANPARFLSPTVRASHHHHHH***** |
| **SpyTagged** *Bacillus licheniformis* α-amylase **(Sp**BLA) | M**AHIVMVDAYKPTK**GGGANLNGTLMQYFEWYMPNDGQHWKRLQNDSAYLAEHGITAVWIPPAYKGTSQADVGYGAYDLYDLGEFHQKGTVRTKYGTKGELQSAIKSLHSRDINVYGDVVINHKGGADATEDVTAVEVDPADRNRVISGEVRIKAWTHFHFPGRGSTYSDFKWHWYHFDGTDWDESRKLNRIYKFQGKAWDWEVSNEFGNYDYLMYADIDYDHPDVVAEIKRWGTWYANELQLDGFRLDAVKHIKFSFLRDWVNHVREKTGKEMFTVAEYWSYDLGALENYLNKTNFNHSVFDVPLHYQFHAASTQGGGYDMRKLLNSTVVSKHPLKAVTFVDNHDTQPGQSLESTVQTWFKPLAYAFILTRESGYPQVFYGDMYGTKGDSQREIPALKHKIEPILKARKQYAYGAQHDYFDHHDIVGWTREGDSSVANSGLAALITDGPGGAKRMYVGRQNAGETWHDITGNRSEPVVINSEGWGEFHVNGGSVSIYVQR* |
| **SpyTagged** *Bacillus licheniformis* α-amylase bearing His6 tag **(Sp**BLA-H6) | M**AHIVMVDAYKPTK**GGGANLNGTLMQYFEWYMPNDGQHWKRLQNDSAYLAEHGITAVWIPPAYKGTSQADVGYGAYDLYDLGEFHQKGTVRTKYGTKGELQSAIKSLHSRDINVYGDVVINHKGGADATEDVTAVEVDPADRNRVISGEVRIKAWTHFHFPGRGSTYSDFKWHWYHFDGTDWDESRKLNRIYKFQGKAWDWEVSNEFGNYDYLMYADIDYDHPDVVAEIKRWGTWYANELQLDGFRLDAVKHIKFSFLRDWVNHVREKTGKEMFTVAEYWSYDLGALENYLNKTNFNHSVFDVPLHYQFHAASTQGGGYDMRKLLNSTVVSKHPLKAVTFVDNHDTQPGQSLESTVQTWFKPLAYAFILTRESGYPQVFYGDMYGTKGDSQREIPALKHKIEPILKARKQYAYGAQHDYFDHHDIVGWTREGDSSVANSGLAALITDGPGGAKRMYVGRQNAGETWHDITGNRSEPVVINSEGWGEFHVNGGSVSIYVQRHHHHHH* |
| **SnoopTagged** *Aequorea victoria* green fluorescent protein bearing His6 tag **(Sn**GFP-H6) | M**GKLGDIEFIKVNK**GSGSGSGSKGEELFTGVVPILVELDGDVNGHKFSVSGEGEGDATYGKLTLKFICTTGKLPVPWPTLVTTLTYGVQCFSRYPDHMKRHDFFKSAMPEGYVQERTIFFKDDGNYKTRAEVKFEGDTLVNRIELKGIDFKEDGNILGHKLEYNYNSHNVYIMADKQKNGIKVNFKIRHNIEDGSVQLADHYQQNTPIGDGPVLLPDNHYLSTQSALSKDPNEKRDHMVLLEFVTAAGITHGMDELYKHHHHHH* |
| **SnoopTagged** *Bacillus licheniformis* α-amylase bearing His6 tag **(Sn**BLA-H6) | M**GKLGDIEFIKVNK**GSGSGSGANLNGTLMQYFEWYMPNDGQHWKRLQNDSAYLAEHGITAVWIPPAYKGTSQADVGYGAYDLYDLGEFHQKGTVRTKYGTKGELQSAIKSLHSRDINVYGDVVINHKGGADATEDVTAVEVDPADRNRVISGEVRIKAWTHFHFPGRGSTYSDFKWHWYHFDGTDWDESRKLNRIYKFQGKAWDWEVSNEFGNYDYLMYADIDYDHPDVVAEIKRWGTWYANELQLDGFRLDAVKHIKFSFLRDWVNHVREKTGKEMFTVAEYWSYDLGALENYLNKTNFNHSVFDVPLHYQFHAASTQGGGYDMRKLLNSTVVSKHPLKAVTFVDNHDTQPGQSLESTVQTWFKPLAYAFILTRESGYPQVFYGDMYGTKGDSQREIPALKHKIEPILKARKQYAYGAQHDYFDHHDIVGWTREGDSSVANSGLAALITDGPGGAKRMYVGRQNAGETWHDITGNRSEPVVINSEGWGEFHVNGGSVSIYVQRHHHHHH* |
| **SdyTagged** *Aequorea victoria* green fluorescent protein bearing His6 tag **(Sd**GFP-H6) | M**DPIVMIDNDKPIT**GSGSGSGSKGEELFTGVVPILVELDGDVNGHKFSVSGEGEGDATYGKLTLKFICTTGKLPVPWPTLVTTLTYGVQCFSRYPDHMKRHDFFKSAMPEGYVQERTIFFKDDGNYKTRAEVKFEGDTLVNRIELKGIDFKEDGNILGHKLEYNYNSHNVYIMADKQKNGIKVNFKIRHNIEDGSVQLADHYQQNTPIGDGPVLLPDNHYLSTQSALSKDPNEKRDHMVLLEFVTAAGITHGMDELYKHHHHHH* |
| **SdyTagged** *Bacillus licheniformis* α-amylase bearing His6 tag **(Sd**BLA-H6) | M**DPIVMIDNDKPIT**GSGSGSGANLNGTLMQYFEWYMPNDGQHWKRLQNDSAYLAEHGITAVWIPPAYKGTSQADVGYGAYDLYDLGEFHQKGTVRTKYGTKGELQSAIKSLHSRDINVYGDVVINHKGGADATEDVTAVEVDPADRNRVISGEVRIKAWTHFHFPGRGSTYSDFKWHWYHFDGTDWDESRKLNRIYKFQGKAWDWEVSNEFGNYDYLMYADIDYDHPDVVAEIKRWGTWYANELQLDGFRLDAVKHIKFSFLRDWVNHVREKTGKEMFTVAEYWSYDLGALENYLNKTNFNHSVFDVPLHYQFHAASTQGGGYDMRKLLNSTVVSKHPLKAVTFVDNHDTQPGQSLESTVQTWFKPLAYAFILTRESGYPQVFYGDMYGTKGDSQREIPALKHKIEPILKARKQYAYGAQHDYFDHHDIVGWTREGDSSVANSGLAALITDGPGGAKRMYVGRQNAGETWHDITGNRSEPVVINSEGWGEFHVNGGSVSIYVQRHHHHHH* |

**Appendix S1.** Experimental section

**Plasmid construction strategy**

To construct pCOLASolo-1_SpyTag-OpdA, the gene encoding SpyTag-OpdA from pET14b_SpyTag-OpdA-His6 was PCR-amplified using primers NcoI-XhoI-SpyTag and AvrII-STOP-OpdA, which also introduce NcoI and XhoI restriction sites before the start codon, and AvrII after the stop codon. The resulting PCR product and vector pCOLADuet-1 were digested with NcoI and AvrII and ligated, which resulted in plasmid pCOLASolo-1_SpyTag-OpdA. Likewise, to construct His6-tagless SpyTagged BLA, the SpyTag-BLA cDNA from plasmid pET14b_SpyTag-BLA-His6 was amplified with primers NcoI-XhoI-SpyTag and AvrII-STOP-BLA and the resulting PCR product cloned into the NcoI/AvrII sites of vector pCOLADuet-1. The resulting plasmid was named pCOLASolo-1_SpyTag-BLA. Plasmid pCOLASolo-1_SpyTag-GFP was generated by amplifying the SpyTag-GFP cDNA from plasmid pET14b_SpyTag-GFP-His6 using primers NcoI-XhoI-SpyTag and AvrII-STOP-GFP. The resulting PCR product and pCOLASolo-1_SpyTag-OpdA were digested with XhoI and AvrII and ligated. The resulting plasmid was designated as pCOLASolo-1_SpyTag-GFP. All the inserts were confirmed by ABI DNA sequencing prior transformation into appropriate sphere and protein production strains.

To generate pET14b_SpyCatcher-PhaC-SnoopCatcher, gene encoding SnoopCatcher was first amplified from pBluescript_II_SK(+)_SdyCatcher-SnoopCatcher using primers XhoI-SnoopCatcher and BamHI-STOP-SnoopCatcher into XhoI/BamHI sites of plasmid pET14b_SpyCatcher_PhaC_linker_SpyCatcher, which resulted in pET14b_SpyCatcher-PhaC-SnoopCatcher. SdyCatcher cDNA amplified from pBluescript_II_SK(+)_SdyCatcher-SnoopCatcher using primers SpeI-START-SdyCatcher and AvrII-SdyCatcher was then ligated into pET14b_SpyCatcher-PhaC-SnoopCatcher at SpeI/AvrII restriction sites, and thereby constructing pET14b_SdyCatcher-PhaC-SnoopCatcher. To create pET14b_SpyCatcher-PhaC-SnoopCatcher, SnoopCatcher cDNA was first amplified from pBluescript_II_SK(+)_SdyCatcher-SnoopCatcher using primers SpeI-START-SnoopCatcher and AvrII-SnoopCatcher into SpeI/AvrII sites of plasmid pET14b_SpyCatcher_PhaC_linker_SpyCatcher, which resulted in pET14b_SnoopCatcher-PhaC-SpyCatcher. Gene encoding SdyCatcher amplified from pBluescript_II_SK(+)_SdyCatcher-SnoopCatcher using primers XhoI-SdyCatcher and BamHI-STOP-SdyCatcher was then ligated into pET14b_SnoopCatcher-PhaC-SdyCatcher at XhoI/BamHI restriction sites, and thereby constructing pET14b_ SnoopCatcher-PhaC- SdyCatcher.

For construction of plasmid pET14b_SnoopTag-linker-GFP-His6, we inserted the SnoopTag-GFP cDNA PCR-amplified from pET14b_PhaC_linker_GFP using primers SpeI-START-SnoopTag-linker-GFP and **SpyTag-GFP-His6_RVR**, into the SpeI and BamHI digested pET14b_SpyTag-GFP-His6. The resulting plasmid was named as pET14b_SnoopTag-L-GFP-His6. Likewise, to create pET14b_SnoopTag-BLA-His6, the *bla* gene from plasmid pET14b-BLAphaC was amplified with primers SpeI-START-SnoopTag-linker-BLA and **SpyTag-BLA-His6_RVR** and by cloning the resulting PCR product into SpeI/BamHI sites of vector pET14b_SpyTag-GFP-His6. The resulting plasmid was named pET14b_SnoopTag-L-BLA-His6. Plasmid pET14b_SdyTag-L-GFP-His6 was constructed by inserting the *SdyTag-gfp* gene PCR-amplified from pET14b_PhaC_linker_GFP using primers SpeI-START-SdyTag-linker-GFP and XhoI-STOP-His6-GFP and ligated into the SpeI and XhoI digested pET14b_C2. The resulting plasmid was named as pET14b_SdyTag-L-GFP-His6. To produce pET14b_SdyTag-L-BLA-His6, the *bla* gene from plasmid pET14b-BLAphaC was amplified with primers SpeI-START-SdyTag-linker-BLA and XhoI-STOP-His6-BLA and by cloning the resulting PCR product into SpeI/XhoI sites of vector pET14b_C2. The resulting plasmid was named pET14b_SdyTag-L-BLA-His6.

| **Table S5.** Protein identification by liquid chromatography-tandem mass spectrometry (LC−MS/MS). | | | | |
| --- | --- | --- | --- | --- |
| Process | Fusion protein | Amino acid coverage (%) | Peptide fragments identified by LC-MS/MS. | Remark |
| N/A | **SpyCatcher-**PhaC**-SpyCatcher** (**S**P**S** only) | 73.9% | **R36-R51, T57-K68, Y77-K109**, S136-R194, D198-K210, F235-R261, F266-R289, N328-K354, Y370-I418, D426-Y560, V572-K682, A709-**K758, R762-S780, T783-K794, Y803-K835.** | From purified SP-S |
| N/A | **SpyCatcher-**PhaC (**S**P only) | 74.7% | **R36-K68, Y77-K109,** V141-R194, D198-K210, F235-R300, I315-K354, Y370-R419, D427-R562, V572-K638, S642-A681, K723-N734. | From purified SPS-S |
| N/A | *Ralstonia eutropha* PhaC (WT only) | 83.9% | S19-R103, T112-R144, I147-R183, N188-R195, K197-K237, Y253-R302, D309-K521, S525-G563. | From purified WT-S |
| 1 | **S**P only | 70.5% | **R36-K99**, S136-R194, D198-R220, F235-R300, I315-K354, Y370-R419, D426-R562, V572-K638, S642-K682, K723-N734. | Unbound SP on SP-S. |
| 1 | **SpGFP**-**S**P ligated protein (**SpGFP**-**S**P-L) | 62.5% | **G14-H40, L68-R88, L156-D170, I182-K224, R230-K253, T310-V320, Y330-G361,** V394-K435, D451-K463, F488-K512, F522-R542, I568-K607, Y623-R672, D679-R815, T830-K871, F874-K891, S895K935, K976-N987. |  |
| 1 | **SpOpdA**-**S**P ligated protein (**SpOpdA**-**S**P-L) | 46.9% | **P38-F60, A87-R103, A114-R134, S137-R147, A185-A240, M250-R270, I291-R326,** V501-R516, D558-K570, F595-K619, F626-R649, N688-K714, Y730-R743, D761-I778, D786-R850, G867-R922, L936-K978, K1083-N1094. |  |
| 1 | **SpBLA**-**S**P ligated protein (**SpBLA**-**S**P-L) | 55.3% | **R41-K87, G124-R142, W172-R186, A198-R246, E272-R322, A337-K387, Q410-K453, Q460-R500,** V641-R656, D698-K710, F735-K759, F766-R789, N828-K854, Y870-R919, D926-R1062, L1076-K1118, F1121-K1138, K1223-N1234. |  |
| 1 | **Sp**BLA (**S**P) | 72.4% | **G15-Q86, D111-V145, W172-R186, A198-R246, T269-R320, A337-K387, K409-K453, Q460-R500.** | Unbound SpBLA after mixing with SP |
| 1 | **S**P**S** only | 55.2% | **R36-R51, T57-K67, Y77-K109,** V141-R156, D198-K210, F235-K259, F266-R289, N328-K354, Y370-R419, D426-R490, G507-K533, L576-K618, A709**-K758, R762-R777, T783-K794, Y803-K838.** | Unbound SPS on SPS-S. |
| 1 | **SpGFP**-**S**P**S** ligated protein (**SpGFP**-**S**P**S**-Ls) | 68.4% | **G15-K42, L69-R89, L157-A170, H185-S224, R231-K254, R290-R305, T311-Q320, Y331-K363,** S390-R448, D452-R474, F489-A555, I569-K608, Y624-R673, D680-K892, S896-K936, A963-**K1012, R1016-L1030, T1037-K1048, Y1056-K1089, G1112-K1139, L1166-R1186**, **L1254-K1269, H1282-K1322, D1329-K1351.** | Protein ligation with either N- or C- terminus SpyCatcher |
| 1 | **SpGFP**-**S**P**S** ligated protein (**SpGFP**-**S**P**S**-L) | 37.2% | **G15-K42, L69-R89, I183-K225, D232-K254, T311-Q320,** D452-K464, F489-R499, F520-R543, N582-K608, Y624-R673, I687-A708, E745-K787, LTV830-A870, A963-R974, **T1037-K1048, G1112-K1139, L1166-R1186, K1279-L1320, D1329-K1351.** | Protein ligation with both N- or C- terminus SpyCatcher |
| 1 | **SpOpdA**-**S**P**S** ligated proteins (**SpOpdA**-**S**P**S**-Ls) | 51.6% | **G37-F60, A87-G102, A114-R134, D203-A240, M250-R270, I291-R314, E333-R351, R396-R411, Y437-K469,** V501-R516, D558-K570, F595-DAK619, F626-R649, N688-K714, Y730-R775, D786-R850, G867-K893, L936-K978, A1069-**K1118, R1122-R1137, Y1163-K1195, P1241-R1265, A1290-R1306, A1317-R1337, S1340-R1350, D1406-R1444, M1453-R1473, I1494-R1517, E1536-R1554.** | Protein ligation with either N- or C- terminus SpyCatcher |
| 1 | **SpOpdA**-**S**P**S** ligated protein (**SpOpdA**-**S**P**S**-L) | 38.2% | **A87-R103, A114-F145, A185-A240, M250-R270, I291-R314,** V501-R516, F595-K619, F626-R649, N689-K714, Y730-I778, D786-R815, G867-K893, L936-K978, A1069**-K1118, A1290-R1306, A1317-R1337, A1398-R1444, M1453-PR1473, I1494-R1517.** | Protein ligation with both N- or C- terminus SpyCatcher |
| 1 | **SpBLA**-**S**P**S** ligated proteins (**SpBLA**-**S**P**S**-Ls) | 58.1% | **R41-Q86, D111-A140, A154-R163, W172-R186, A198-R246, E272-R322, A337-K387, E431-K453, Q460-R500, R536-L550,** V641-R656, D698-K710, F735-K759, F766-R789, I815-K854, Y870-R919, D926-K1033, T1064-K1118, F1121-K1138, A1209**-K1258, R1262-E1275, R1384-K1430, D1454-R1485**, **A1497-R1506, W1515-R1529, A1541-R1589, E1615-R1665, A1680-K1730, E1774-K1796, Q1803-R1843.** | Protein ligation with either N- or C- terminus SpyCatcher |
| 1 | **SpBLA**-**S**P**S** ligated protein (**SpBLA**-**S**P**S**-L) | 28.6% | **R41-K64, G124-A140, W232-R246, E272-K293, A337-T370, E431-K453,**  A746-K759, F766-R789, N828-K854, N899-R919, D927R955, G1007-K1033, L1076-K1118, A1209-**K1258, R1384-Y1406, G1467-R1485, W1575-R1589, E1615-K1636, V1681-R1714, E1774-D1790**. | Protein ligation with both N- or C- terminus SpyCatcher |
| 1 | **SpBLA** (**S**P**S**) | 72.6% | **G15-K86, D111-V145, W172-R186, A198-R246, T269-R320, A337-K387, Q410-K453, Q460-R500.** | Unbound SpBLA after mixing with SPS |
| 2 | **S**P only | 66.9% | **R36-L50, T57-K68, Y77-K109,** V141-R156, I183-R194, F235-K259, F266-SLR300, I268-K354, Y370-R419, D426-R562, L576-K618, F621-K638, S666-A681, K723-N734. | Unbound SP on SP-S. |
| 2 | **SpGFP**-**S**P ligated protein (**SpGFP**-**S**P-L) | 53.4% | **G15-K42, L157-D171, I183-K225, R231-K254, D291-R305, T311-V321,** V395-R410, D452-FK464, F489-K513, F520-R554, I569-K608, Y624-R673, D680-R816, L830-K872, F875-K892, K977-N988. |  |
| 2 | **SpOpdA**-**S**P ligated protein (**SpOpdA**-**S**P-L) | 51.2% | **G37-F60, A87-AR103, A114-R134, S137-R147, A185-A240, M250-R270, I291-R326, E333-R348,** V501-R516, D558-K570, F595-K619, A627-R649, N688-K714, Y730-R779, D786-R922, L936- K998. |  |
| 2 | **SpBLA**-**S**P ligated protein (**SpBLA**-**S**P-L) | 64.6% | **G15-K87, G124-R142, A154-R163, W172-R186, A198-R246, E272-R322, A337-K387, Y411-AK453, Q460-R500, T557-K568,** V641-R656, I683-K635, F735-K759, F766-R800, N828-K854, Y870-R919, D926-K1118, F1121-K1138, S1142-K1182. |  |
| 2 | **S**P**S** only (Process 1) | 76.6% | **R36-R51, T57-K68, Y77-K109,** V141-K208, F235-R261, I264-R300, N305-R312, I315-K354, Y370-R419, D426-R562, V572-K638, S642-K682, A709**-K758, R762-R777, T783-K794, Y803-K835.** | Unbound SPS on SPS-S after mixing. |
| 2 | **SpGFP**-**S**P**S** ligated protein (**SpGFP**-**S**P**S**-Ls) | 53.6% | **G15-K42, L69-Y90, I183-254, R290-R305, T311-K322, Y331-K363,** V395-R410, I437-R448, D452-K464, F489-K513, F520-R554, N582-K605, Y624-R673, I687-K787, L830-K872, A963-**K1012, R1016-K1048, Y1057-K1089, G1112-K1139, L1166-R1186, I1280-K1322, D1329-K1351.** | Protein ligation with either N- or C- terminus SpyCatcher |
| 2 | **SpGFP**-**S**P**S** ligated protein (**SpGFP**-**S**P**S**-L) | 36.1% | **G15-K42**, **T113-K123, I183-K225, D232-K254,** I437-R448, F489-K513, F520-R554, N582-K608, Y624-R637, N653-R673, I687-R709, G761-K787, L830-K872, A963**-K1012, G1112-K1139, T1210-K1220, I1280-K1322, D1329-K1351.** | Protein ligation with both N- or C- terminus SpyCatcher |
| 2 | **SpOpdA**-**S**P**S** ligated proteins (**SpOpdA**-**S**P**S**-Ls) | 41.5% | **A114-R134, S137-R147, A185-A240, M250-R270, I291-R314, D397-R411,** V501-R516, D558-K570, F595-K619, F626-R649, I675-K714, H744-R779, D786-R815, G867-R922, L936-K978, F981-K998, A1069**-K1118, D1123-R1137, A1317-R1337, S1340-R1350, A1388-R1444, M1453-R1473, I1494-R1517.** | Protein ligation with either N- or C- terminus SpyCatcher |
| 2 | **SpOpdA**-**S**P**S** ligated protein (**SpOpdA**-**S**P**S**-L) | 44.6% | **G37-R62, A87-R103, A114-R147, A185-A240, M250-R270, E333-R351, R396-R411,** V501-R554, F595-K619, F626-R649, N688-K714, Y730-R779, D786-R815, L936-K978, F981-K998, A1069-**K1118, R1122-R1137, G1240-R1260, A1290-R1306, A1317-R1350, A1388-R1441, M1453-R1473, E1536-R1554.** | Protein ligation with both N- or C- terminus SpyCatcher |
| 2 | **SpBLA**-**S**P**S** ligated proteins (**SpBLA**-**S**P**S**-Ls) | 43.7% | **G65-K87, G124-R142, A198-R246, E272-K293, A337-K387, E431-K453, S474-R500, T557-K568,** V641-R656, F735-K759, F766-R800, I768-K854, H884-R919, D926-R990, G1007-K1033, L1076-K1118, F1121-K1137, A1209-**K1258, T1283-K1294, G1408-K1430, G1467-R1485, A1541-R1589, E1615-K1636, A1680-K1730, E1774-K1796, S1817-R1843.** | Protein ligation with either N- or C- terminus SpyCatcher |
| 2 | **SpBLA**-**S**P**S** ligated protein (**SpBLA**-**S**P**S**-L) | 46.7% | **R41-K87, G124-R142, A198-R246, E272-R322, A337-R371, E431-K453, S474-R500,** V641-R656, D698-K710, F735-K759, F766-R800, I815-K854, Y870-R919, D926-R955, G1007-K1033, L1076-K1118, A1209**-K1258, R1262-R1276, R1384-K1430, G1467-R1485, A1541-R1589, E1615-R1665, A1680-R1714, E1774-K1796, S1817-R1843.** | Protein ligation with both N- or C- terminus SpyCatcher |
| 3 | **S**P only | 76.4% | **R36-L50, T57-K68, Y77-K109,** S136-R194, D198-R220, F235-R261, I264-R300, I268-D353, Y370-R419, D426-K682, K723-N734. | Unbound SP on SP-S. |
| 3 | **SpGFP**-**S**P ligated protein (**SpGFP**-**S**P-L) | 55.9% | **G14-K41, L68-R88, A125-R137, L156-K171, I182-K224, D231-K253, D290-R304, Y330-K362,** V394-R409, D451-K463, F488-K512, F519-R542, I521-K607, Y623-R672, D679-R815, L829-K817, K976-N987. |  |
| 3 | **SpGFP** (**S**P) | 61.0% | **G15-L58, F62-R89, A126-R138, L157-K172, I183-K225, R231-K254.** | Unbound SpGFP after mixing with SP |
| 3 | **SpOpdA**-**S**P ligated protein (**SpOpdA**-**S**P-L) | 46.2% | **G37-A73, A87-R103, A114-R134, S137-R147, A185-A240, M250-R270, I291-R326,** V501-R516, D558-K570, F595-K619, F626-R649, N688-K714, Y730-R743, N316-R779, D786-R850, G867-R922, L936-TK978, K1083-N1094. |  |
| 3 | **SpOpdA** (**S**P) | 72.8% | **G18-R62, A87-R106, A114-R134, S137-R159, A171-A240, M250-R270, A276-R285, I291-R326, E333-R351.** | Unbound SpOpdA after mixing with SP |
| 3 | **SpBLA**-**S**P ligated protein (**SpBLA**-**S**P-L) | 53.9% | **R41-K87, A198-R246, E272-R322, A337-K387, E431-K453, S474-R500, R536-R550,** V641-R656, D698-K710, F735-K759, F766-R800, N828-K854, Y871-R919, D926-R1062, L1076-K1118, F1121-K1138, L1224-N1234. |  |
| 3 | **SpBLA** (**S**P) | 75.0% | **G15-K87, D111-R144, I152-R163, W172-K187, A198-K251, E272-R322, A337-T386, Y411-R454, Q460-R500.** | Unbound SpBLA after mixing with SP |
| 3 | **S**P**S** only (Process 1) | 77.3% | **R36-K68, Y77-K109**, V141-R194, D198-K210, F235-R261, I264-R300, I315-K354, Y371-R419, D426-K638, S642-K682, A709**-K758, R762-R777, T783-K794, Y803-K835.** | Unbound SPS on SPS-S after mixing. |
| 3 | **SpGFP**-**S**P**S** ligated protein (**SpGFP**-**S**P**S**-Ls) | 51.2% | **G15-K42, L69-Y90, I183-K225, D232-K254, R290-K322, Y311-K363,** V395-R410, D452-K464, F489-K513, F520-R543, N582-K608, Y624-R673, D680-R744, G761-K787, L830-K872, A963-**K1012, R1016-R1031, T1037-K1048, Y1057-K1089, G1112-K1139, L1166-R1186, I1280-K1322, D1329-K1351.** | Protein ligation with either N- or C- terminus SpyCatcher |
| 3 | **SpGFP**-**S**P**S** ligated protein (**SpGFP**-**S**P**S**-L) | 50.3% | **G15-K42, L69-R89, T113-R138, H185-K225, D232-K254, R290-R305, T311-K322,** V395-R410, D452-K464, F489-K513, F520-R554, N582-K608, Y624-R673, D680-R709, E745-K787, L830-872, F875-K892, A963-**K1012, R1016-R1031, T1037-K1048, G1112-K1139, L1166-R1186, T1210-R1235, H1282-K1320, D1329-K1351.** | Protein ligation with both N- or C- terminus SpyCatcher |
| 3 | **SpGFP** (**S**P**S**) | 67.7% | **G15-K57, F62-R89, A126-R138, L157-K172, I183-K254.** | Unbound SpGFP after mixing with SPS |
| 3 | **SpOpdA**-**S**P**S** ligated proteins (**SpOpdA**-**S**P**S**-Ls) | 60.3% | **G18-R62, A87-R103, A114-R134, S137-R147, A185-A240, M250-R270, I291-R326, E333-R351, R396-R411, T417-K428, Y437-K469,** V501-R516, D558-FK570, F595-K619, F626-R660, N688-K714, Y730-R779, D786-K893, L936-K978, A1069**-K1118, R1122-R1137, T1143-K1154, Y1163-K1195, G1221-1265, A1290-R1306, A1317-R1337, S1340-R1350, A1390-A1443, M1453-R1473, I1494-R1529, E1536-R1554.** | Protein ligation with either N- or C- terminus SpyCatcher |
| 3 | **SpOpdA**-**S**P**S** ligated protein (**SpOpdA**-**S**P**S**-L) | 40.7% | **A87-R103, A114-R134, S137-R147, D203-A240, M250-R270, I291-R326, E333-R351, V501-R516, F595-R605, F626-R649, N688-K714, Y730-R743, N758-R779, D786-R815, E851-R922, L936-K978, A1069-K1118, R1122-R1137, A1290-R1306, A1317-R1337, S1340-R1350, D1406-R1444, M1453-R1473, I1494-R1529, E1536-R1554.** | Protein ligation with both N- or C- terminus SpyCatcher |
| 3 | **SpOpdA** (**S**P**S**) | 75.6% | **G18-R62, A87-R106, A114-R134, S137-R159, K164-A240, G242-R270, I291-R324, E333-R351.** | Unbound SpOpdA after mixing with SPS |
| 3 | **SpBLA**-**S**P**S** ligated proteins (**SpBLA**-**S**P**S**-Ls) | 46.6% | **R41-K87, W172-R186, A198-R246, E272-R322, A337-T370, E431-K453, S474-R500, T557-K568,** V641-R656, F735-K759, F766-R789, N828-K854, Y870-R919, D926-R990, G1007-K1033, L1076-K1118, A1209-**K1258, T1283-K1294, R1384-K1430, W1515-R1529, A1541-R1589, E1615-R1665, A1680-T1713, E1774-K1796, S1817-R1843.** | Protein ligation with either N- or C- terminus SpyCatcher |
| 3 | **SpBLA** (**S**P**S**) | 73.8% | **G15-K87**, **D111-R142, A154-R163, W172-K187, A198-R246, E272-R322, A337-K387, K409-R454, Q460-R500.** | Unbound SpBLA after mixing with SPS |

**Table S6.** Amount of various functionalized SpyCatcher-coated PHA spheres produced using process 1. SpGFP-SP-S, SpGFP-SP ligated protein-displaying PHA spheres; SpOpdA-SP-S, SpOpdA-SP ligated protein-displaying PHA spheres; SpBLA-SP-S, SpBLA-SP ligated protein-displaying PHA spheres; SP-S, SP fusion protein-displaying PHA spheres; SpGFP-SPS-S, SpGFP-SPS ligated protein-displaying PHA spheres; SpOpdA-SPS-S, SpOpdA-SPS ligated protein-displaying PHA spheres; SpBLA-SPS-S, SpBLA-SPS ligated protein-displaying PHA spheres; SPS-S, SPS fusion protein-displaying PHA spheres.

| **Sample** | **g wet sphere per L of culture** | **g cell mass per L** |
| --- | --- | --- |
| SpGFP-SP-S | 1.87 | 6.01 |
| SpOpdA-SP-S | 2.71 | 5.56 |
| SpBLA-SP-S | 3.73 | 5.97 |
| SP-S (negative plasmid control) | 1.37 | 6.04 |
| SpGFP-SPS-S | 2.22 | 6.66 |
| SpOpdA-SPS-S | 2.40 | 5.57 |
| SpBLA-SPS-S | 3.07 | 5.28 |
| SPS-S (negative plasmid control) | 1.75 | 5.76 |

**Table S7.** Amount of various functionalized SpyCatcher-coated PHA spheres produced using process 3. SpGFP-SP-S, SpGFP-SP ligated protein-displaying PHA spheres; SpOpdA-SP-S, SpOpdA-SP ligated protein-displaying PHA spheres; SpBLA-SP-S, SpBLA-SP ligated protein-displaying PHA spheres; SP-S, SP fusion protein-displaying PHA spheres; SpGFP-SPS-S, SpGFP-SPS ligated protein-displaying PHA spheres; SpOpdA-SPS-S, SpOpdA-SPS ligated protein-displaying PHA spheres; SpBLA-SPS-S, SpBLA-SPS ligated protein-displaying PHA spheres; SPS-S, SPS fusion protein-displaying PHA spheres.

| **Sample** | **g wet sphere collected after sphere isolation step** | **g cell containing SpyCatcher-coated PHA spheres introduced into the process** |
| --- | --- | --- |
| SpGFP-SP-S | 0.65 | 2.00 |
| SpOpdA-SP-S | 0.46 | 2.00 |
| SpBLA-SP-S | 0.72 | 2.00 |
| SP-S (negative plasmid control) | 0.66 | 2.00 |
| SpGFP-SPS-S | 0.78 | 2.00 |
| SpOpdA-SPS-S | 0.89 | 2.00 |
| SpBLA-SPS-S | 1.08 | 2.00 |
| SPS-S (negative plasmid control) | 0.43 | 2.00 |


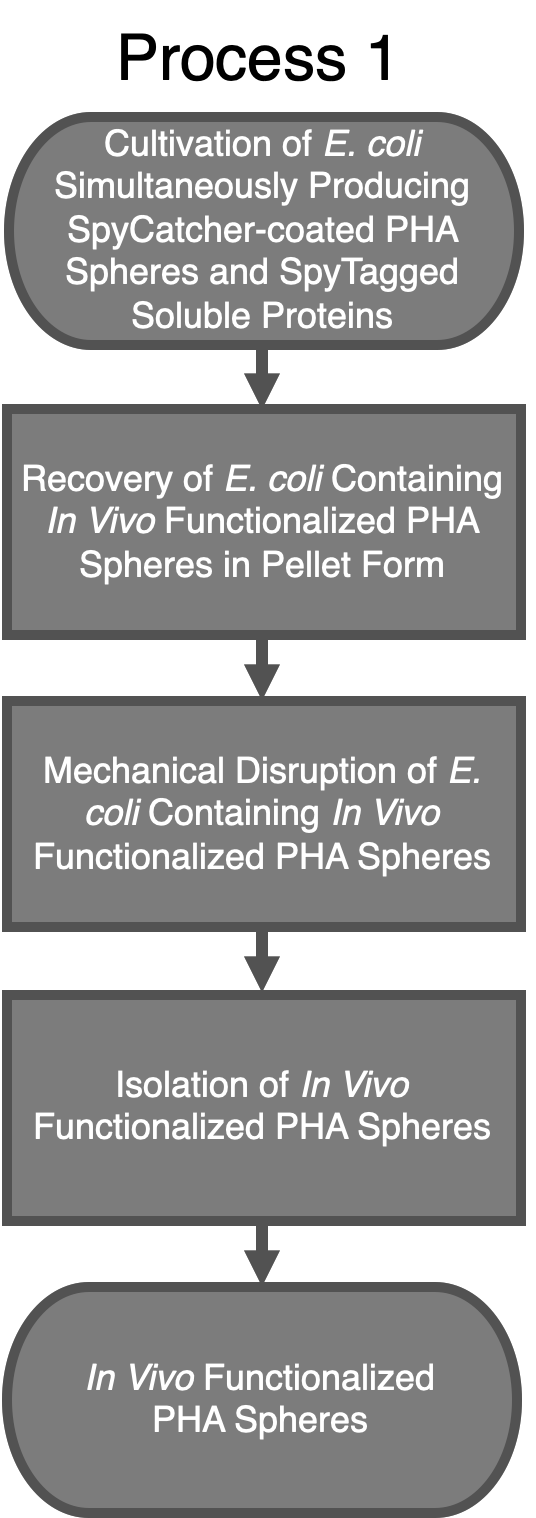


**Figure S1.** Flowchart of *in vivo* functionalization of SpyCatcher-coated PHA spheres using process 1.

**Figure S2.** Flowchart of *ex vivo* functionalization of SpyCatcher-coated PHA spheres using process 2.

**
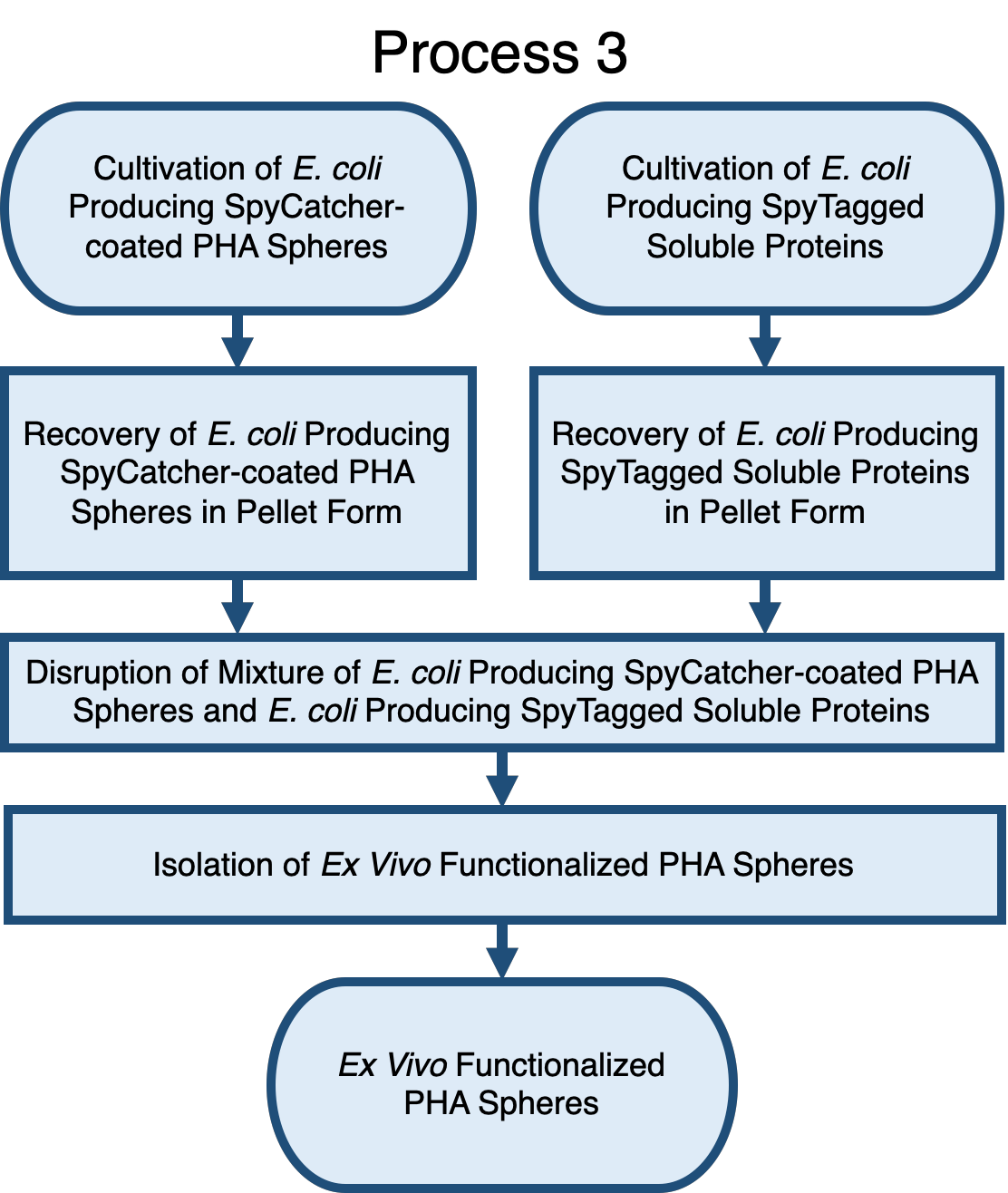
**

**Figure S3.** Flowchart of *ex vivo* functionalization of SpyCatcher-coated PHA spheres using process 3.

**Figure S4.** Three possible ligated products from functionalized SpyCatcher-PhaC-SpyCatcher fusion protein-displaying PHA spheres (SPS-S) (orange/brown) using SpyTagged proteins. Upon mixture of SPS-S with SpyTagged protein of interest (purple/green), SpyTagged proteins could immobilize on C- or/and N-terminal SpyCatcher domains.


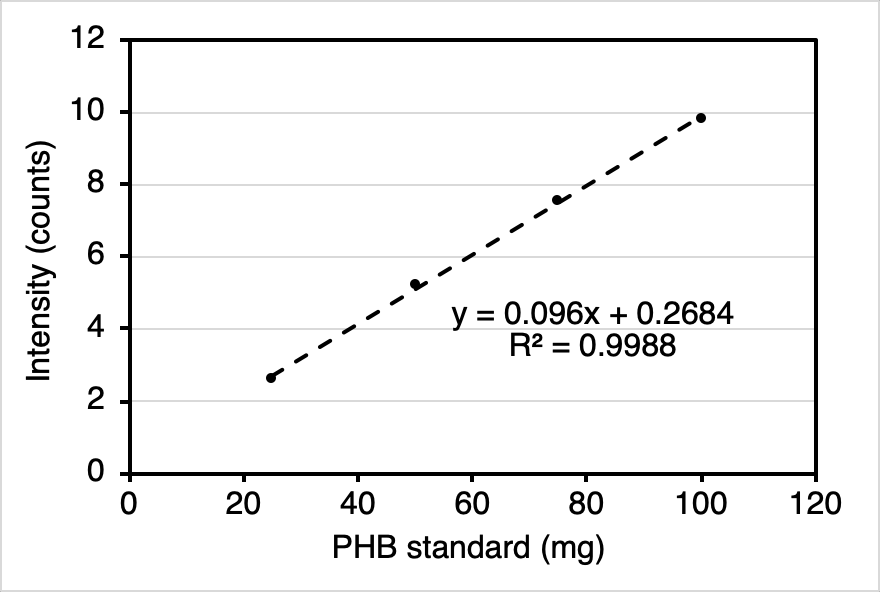


**Figure S5.** Poly(*R*)-3-hydroxybutyrate (PHB) standard curve obtained from GC−MS. Pure PHB as a standard for compositional analysis of SpyCatcher-coated PHA spheres.





**Figure S6.** Whole cell lysate (WCL) of *E. coli* BL21(DE3) containing modular *in vivo* functionalized SpyCatcher-coated PHA spheres (Process 1). **(A)** WCL of *E. coli* BL21(DE3) containing *in vivo* functionalized SP-Ss. **(B)** WCL of *E. coli* BL21(DE3) containing *in vivo* functionalized SPS-Ss. SP-S, SP-displaying PHA spheres; SPS-S, SPS-displaying PHA spheres.





**Figure S7.** Chlorferon standard curve obtained from fluorescence spectroscopy for the OpdA activity assay.





**Figure S8.** Densitometric protein quantification of wild-type PhaC (WT) on PHA spheres relative to BSA standards. **(A)** SDS-PAGE analysis of WT at varying dilution factors. Lane M, Gangnam pre-stained protein ladder; lane 1, BSA (50 ng); lane 2, BSA (100 ng); lane 3, BSA (200 ng); lane 4, BSA (400 ng); lane 5, WT (dilution factor of 6); lane 6, WT (dilution factor of 12); lane 7, WT (dilution factor of 24); lane 8, WT (dilution factor of 47); lane 9, WT (dilution factor of 94). **(B)** BSA standard curve obtained from SDS-PAGE densitometric analysis.





**Figure S9.** Densitometric protein quantification of SpyCatcher-PhaC (SP) fusion protein on PHA spheres relative to BSA standards. **(A)** SDS-PAGE analysis of SP fusion protein at varying dilution factors. Lane M, Gangnam pre-stained protein ladder; lane 1, BSA (50 ng); lane 2, BSA (100 ng); lane 3, BSA (200 ng); lane 4, BSA (400 ng); lane 5, SP fusion protein (dilution factor of 12); lane 6, SP fusion protein (dilution factor of 24); lane 7, SP fusion protein (dilution factor of 47); lane 8, SP fusion protein (dilution factor of 94); lane 9, SP fusion protein (dilution factor of 187). **(B)** BSA standard curve obtained from SDS-PAGE densitometric analysis.





**Figure S10.** Densitometric protein quantification of SpyCatcher-PhaC-SpyCatcher (SPS) fusion protein on PHA spheres relative to BSA standards. **(A)** SDS-PAGE analysis of SPS fusion protein at varying dilution factors**.** Lane M, Gangnam pre-stained protein ladder; lane 1, BSA (50 ng); lane 2, BSA (100 ng); lane 3, BSA (200 ng); lane 4, BSA (400 ng); lane 5, SPS fusion protein (dilution factor of 12); lane 6, SPS fusion protein (dilution factor of 24); lane 7, SPS fusion protein (dilution factor of 47); lane 8, SPS fusion protein (dilution factor of 94); lane 9, SPS fusion protein (dilution factor of 187). **(B)** BSA standard curve obtained from SDS-PAGE densitometric analysis.





**Figure S11.** Densitometric protein quantification of PhaC-OpdA fusion protein on PHA spheres relative to BSA standards. **(A)** SDS-PAGE analysis of PhaC-OpdA fusion protein at varying dilution factors**.** Lane M, Gangnam pre-stained protein ladder; lane 1, BSA (50 ng); lane 2, BSA (100 ng); lane 3, BSA (200 ng); lane 4, BSA (400 ng); lane 5, PhaC-OpdA fusion protein (dilution factor of 6); lane 6, PhaC-OpdA fusion protein (dilution factor of 12); lane 7, PhaC-OpdA fusion protein (dilution factor of 24); lane 8, PhaC-OpdA fusion protein (dilution factor of 47); lane 9, PhaC-OpdA fusion protein (dilution factor of 94). **(B)** BSA standard curve obtained from SDS-PAGE densitometric analysis.





**Figure S12.** Densitometric protein quantification of SpOpdA-SP ligated protein (SpOpdA-SP-L) on PHA spheres relative to BSA standards (Process 1). **(A)** SDS-PAGE analysis of SpOpdA-SP-L at varying dilution factors**.** Lane M, Gangnam pre-stained protein ladder; lane 1, BSA (50 ng); lane 2, BSA (100 ng); lane 3, BSA (200 ng); lane 4, BSA (400 ng); lane 5, SpOpdA-SP-L (dilution factor of 6); lane 6, SpOpdA-SP-L (dilution factor of 12); lane 7, SpOpdA-SP-L (dilution factor of 24); lane 8, SpOpdA-SP-L (dilution factor of 47); lane 9, SpOpdA-SP-L (dilution factor of 94). **(B)** BSA standard curve obtained from SDS-PAGE densitometric analysis.





**Figure S13.** Densitometric protein quantification of SpOpdA-SPS ligated protein (SpOpdA-SPS-L) on PHA spheres relative to BSA standards (Process 1). **(A)** SDS-PAGE analysis of SpOpdA-SPS-L at varying dilution factors**.** Lane M, Gangnam pre-stained protein ladder; lane 1, BSA (50 ng); lane 2, BSA (100 ng); lane 3, BSA (200 ng); lane 4, BSA (400 ng); lane 5, SpOpdA-SPS-L (dilution factor of 6); lane 6, SpOpdA-SPS-L (dilution factor of 12); lane 7, SpOpdA-SPS-L (dilution factor of 24); lane 8, SpOpdA-SPS-L (dilution factor of 47); lane 9, SpOpdA-SPS-L (dilution factor of 94). **(B)** BSA standard curve obtained from SDS-PAGE densitometric analysis.





**Figure S14.** Densitometric protein quantification of SpOpdA-SP ligated protein (SpOpdA-SP-L) on PHA spheres relative to BSA standards (Process 2). **(A)** SDS-PAGE analysis of SpOpdA-SP-L at varying dilution factors**.** Lane M, Gangnam pre-stained protein ladder; lane 1, BSA (50 ng); lane 2, BSA (100 ng); lane 3, BSA (200 ng); lane 4, BSA (400 ng); lane 5, SpOpdA-SP-L (dilution factor of 12); lane 6, SpOpdA-SP-L (dilution factor of 24); lane 7, SpOpdA-SP-L (dilution factor of 47); lane 8, SpOpdA-SP-L (dilution factor of 94); lane 9, SpOpdA-SP-L (dilution factor of 187). **(B)** BSA standard curve obtained from SDS-PAGE densitometric analysis.





**Figure S15.** Densitometric protein quantification of SpOpdA-SPS ligated protein (SpOpdA-SPS-L) on PHA spheres relative to BSA standards (Process 2). **(A)** SDS-PAGE analysis of OpdA-SPS-L at varying dilution factors**.** Lane M, Gangnam pre-stained protein ladder; lane 1, BSA (50 ng); lane 2, BSA (100 ng); lane 3, BSA (200 ng); lane 4, BSA (400 ng); lane 5, SpOpdA-SPS-L (dilution factor of 12); lane 6, SpOpdA-SPS-L (dilution factor of 24); lane 7, SpOpdA-SPS-L (dilution factor of 47); lane 8, SpOpdA-SPS-L (dilution factor of 94); lane 9, SpOpdA-SPS-L (dilution factor of 187). **(B)** BSA standard curve obtained from SDS-PAGE densitometric analysis.





**Figure S16.** Densitometric protein quantification of N-terminally SpyTagged and C-terminally hexahistidine-tagged soluble OpdA (SpOpdA-H6). **(A)** SDS-PAGE analysis of SpOpdA-His6 and BSA standards**.** Lane M, Gangnam pre-stained protein ladder; lane 1, BSA (50 ng); lane 2, BSA (100 ng); lane 3, BSA (200 ng); lane 4, BSA (400 ng); lane 5, BSA (400 ng); lane 6, SpOpdA-H6 (dilution factor of 24). **(B)** BSA standard curve obtained from SDS-PAGE densitometric analysis.

**Figure S17.** Densitometric protein quantification of various combinations of Catcher domains fused PhaC fusion proteins relative to BSA standards. **(A)** SDS-PAGE analysis of various Catcher domain pairs fused to PhaC fusion proteins at varying dilution factors**.** Lane M, Gangnam pre-stained protein ladder; lane 1, BSA (50 ng); lane 2, BSA (100 ng); lane 3, BSA (200 ng); lane 4, BSA (400 ng); lane 5, NPD fusion protein (dilution factor of 94); lane 6, DPN fusion protein (dilution factor of 187); lane 7, PPN fusion protein (dilution factor of 187); lane 8, NPP fusion protein (dilution factor of 187). **(B)** BSA standard curve obtained from SDS-PAGE densitometric analysis.

**Figure S18.** Densitometric protein quantification of various tagged GFP fusion proteins relative to BSA standards. **(A)** SDS-PAGE analysis of different tagged GFP fusion proteins at dilution factor of 38**.** Lane M, Gangnam pre-stained protein ladder; lane 1, BSA (50 ng); lane 2, BSA (100 ng); lane 3, BSA (200 ng); lane 4, BSA (400 ng); lane 5, SpGFP-H6; lane 6, SnGFP-H6; lane 7, SdGFP-H6. **(B)** BSA standard curve obtained from SDS-PAGE densitometric analysis.

**Figure S19.** Densitometric protein quantification of different tagged BLA fusion proteins relative to BSA standards. **(A)** SDS-PAGE analysis of different tagged BLA fusion proteins at dilution factor of 38**.** Lane M, Gangnam pre-stained protein ladder; lane 1, BSA (50 ng); lane 2, BSA (100 ng); lane 3, BSA (200 ng); lane 4, BSA (400 ng); lane 5, SpBLA-H6; lane 6, SnBLA-H6; lane 7, SdBLA-H6. **(B)** BSA standard curve obtained from SDS-PAGE densitometric analysis.


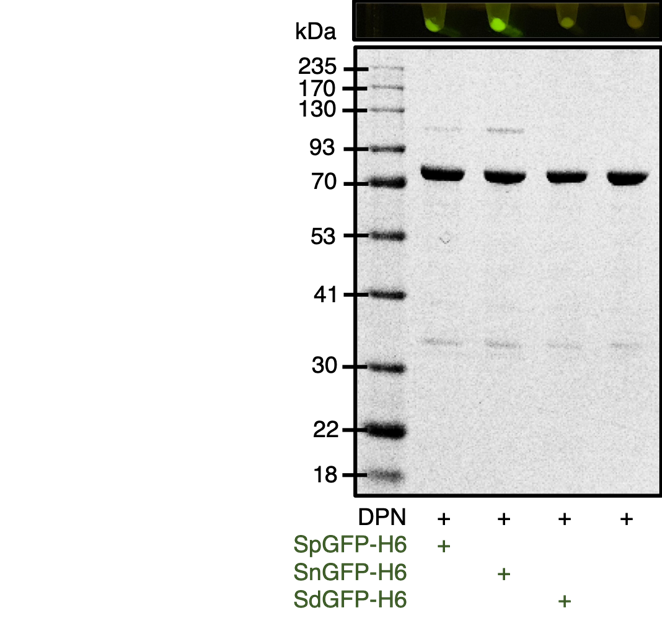


**Figure S20.** Modular functionalization of SdyCatcher-PhaC-SnoopCatcher fusion protein (DPN)-displaying PHA spheres (DPN-S) *in vitro* using various tagged GFPs. Functionalized PHA spheres were visualized by blue light exposure (top) and SDS-PAGE analysis (bottom). SpGFP-H6, SpyTagged GFP bearing His6; SnGFP-H6, SnoopTagged GFP bearing His6; SdGFP-H6, SdyTagged GFP bearing His6.


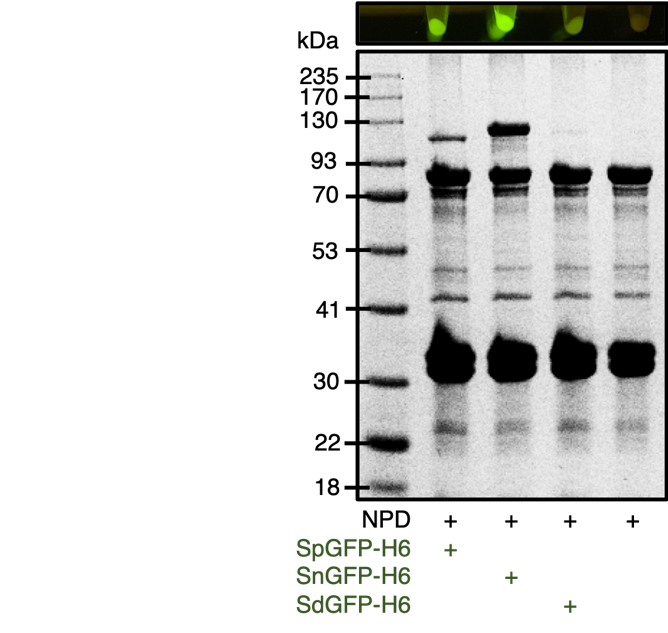


**Figure S21.** Modular functionalization of SnoopCatcher-PhaC-SdyCatcher fusion protein (NPD)-displaying PHA spheres (NPD-S) *in vitro* using various tagged GFPs. Functionalized PHA spheres were visualized by blue light exposure (top) and SDS-PAGE analysis (bottom). SpGFP-H6, SpyTagged GFP bearing His6; SnGFP-H6, SnoopTagged GFP bearing His6; SdGFP-H6, SdyTagged GFP bearing His6.


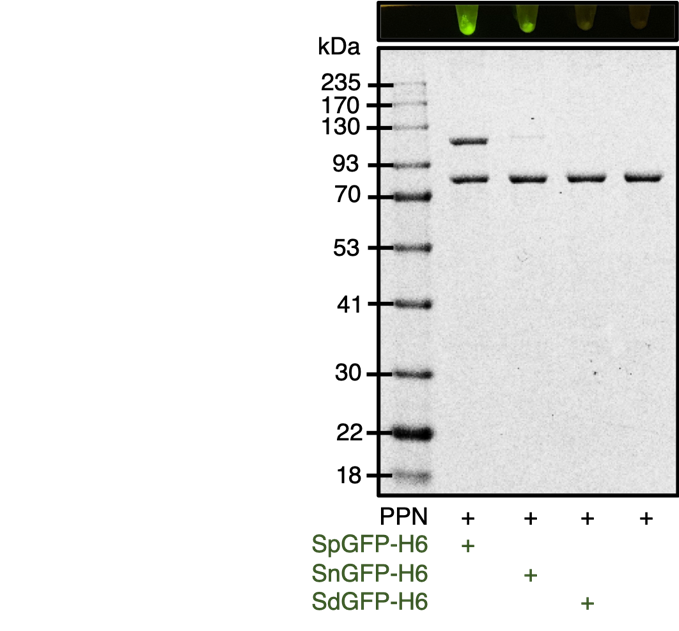


**Figure S22.** Modular functionalization of SpyCatcher-PhaC-SnoopCatcher fusion protein (PPN)-displaying PHA spheres (PPN-S) *in vitro* using various tagged GFPs. Functionalized PHA spheres were visualized by blue light exposure (top) and SDS-PAGE analysis (bottom). SpGFP-H6, SpyTagged GFP bearing His6; SnGFP-H6, SnoopTagged GFP bearing His6; SdGFP-H6, SdyTagged GFP bearing His6.

**Figure S23.** SnoopCatcher-PhaC-SpyCatcher fusion protein-displaying PHA spheres (NPP-S) could react with tagged proteins individually and simultaneously in complex environments *ex vivo* (Process 2). Lane M, Gangnam pre-stained protein ladder; lane 1, functionalized NPP-S prepared using cleared lysate containing SpGFP-H6 after washing; lane 2, functionalized NPP-S prepared using cleared lysate containing SnGFP-H6 after washing; lane 3, functionalized NPP-S prepared using cleared lysate containing both SpGFP-H6 and SnGFP-H6 after washing; lane 4, mixture of NPP-S and cleared lysate containing SpGFP-H6 after incubation and before washing; lane 5, mixture of NPP-S and cleared lysate containing SnGFP-H6 after incubation and before washing; lane 6, mixture of NPP-S and cleared lysate containing both SpGFP-H6 and SnGFP-H6 after incubation and before washing; lane 7, plain NPP-S; lane 8, cleared lysate containing SpGFP-H6; lane 9, cleared lysate containing SnGFP-H6; lane 10, cleared lysate containing SpGFP-H6 and SnGFP-H6.

**References**

Amara, A.A., and Rehm, B.H. (2003). Replacement of the catalytic nucleophile cysteine-296 by serine in class II polyhydroxyalkanoate synthase from Pseudomonas aeruginosa-mediated synthesis of a new polyester: identification of catalytic residues. *Biochem. J.* 374**,** 413-421. doi:

Wong, J.X., and Rehm, B.H. (2018). Design of modular polyhydroxyalkanoate scaffolds for protein immobilization by directed ligation. *Biomacromolecules* 19**,** 4098-4112. doi: 10.1021/acs.biomac.8b01093
